# Supplementary material for: High-temperature electrothermal remediation of multi-pollutants in soil
Source: Nat Commun. 2023 Oct 11;14:6371. doi: 10.1038/s41467-023-41898-z (PMC10567823; doi:10.1038/s41467-023-41898-z)
Supplement: Supplementary file 1 — Supplementary Information [file 41467_2023_41898_MOESM1_ESM.pdf]

Supplementary Information for

High-Temperature Electrothermal Remediation of Multi-Pollutants in Soil

Bing Deng,[1,#,*] Robert A. Carter,[1,#] Yi Cheng,[1,#] Yuan Liu,[2] Lucas Eddy,[1,3] Kevin M. Wyss,[1] Mine G. Ucak-Astarlioglu,[4] Duy Xuan Luong,[1,3] Xiaodong Gao[5,6], Khalil JeBailey,[7] Carter Kittrell,[1] Shichen Xu[1], Debadrita Jana,[5] Mark Albert Torres,[5] Janet Braam,[2] and James M. Tour[1,7,8,9,*]

[1] Department of Chemistry, Rice University, Houston TX, 77005, USA.

[2] Department of BioSciences, Rice University, Houston TX, 77005, USA.

[3] Applied Physics Program, Rice University, Houston TX, 77005, USA.

[4] Geotechnical and Structures Laboratory, U.S. Army Engineer Research & Development Center, Vicksburg MS, 39180, USA.

[5] Department of Earth, Environmental, & Planetary Sciences, Rice University, Houston TX, 77005, USA.

[6] Carbon Hub, Rice University, Houston TX, 77005, USA.

[7] Department of Materials Science and NanoEngineering, Rice University, Houston TX, 77005, USA.

[8] Smalley-Curl Institute, Rice University, Houston TX, 77005, USA.

[9] NanoCarbon Center and the Welch Institute for Advanced Materials, Rice University, Houston TX, 77005, USA.

[#] Those authors contributed equally.

* Corresponding authors: J.M.T. (tour@rice.edu), B.D. (bingdeng@rice.edu)

Supplementary Note 1. Influence of chemical species on removal efficiencies of heavy metals

In the HET process, the elevated temperatures can initiate various reactions, including carbothermic reduction facilitated by the presence of carbon conductive additives, thermal decomposition, and evaporation. Consequently, there are multiple potential pathways for the removal of different heavy metals species: (1) Direct evaporation of metal species; (2) Thermal decomposition of metal species to other species followed by their evaporation; and (3) Carbothermic reduction of metal species to lower-valence-state species followed by their evaporation. Given that the temperature in the HET process can reach up to 3000 °C, all of the above reactions have the potential to occur, providing various pathways for the removal of different heavy metal species.

In this study, we focused on Hg as an example to investigate the impact of speciation on removal efficiency. The Hg contaminants present in the soil primarily exist in the form of Hg(0) and Hg(II) (ref[1]). Depending on the counterions, Hg(II) species can include HgS, HgO, $HgCl_2$, $HgSO_4$, and so on[1]. To understand the potential reactions involved, we conducted a thermodynamic analysis using the software HSC Chemistry 10.

For Hg(0), direct evaporation occurs as represented by:

$Hg(l) = Hg(g)$ (1)

For $HgCl_2$, it can undergo direct evaporation:

$HgCl_2(s) = HgCl_2(g)$ (2)

Alternatively, $HgCl_2$ can decompose to Hg (ref[2]) followed by evaporation:

$HgCl_2(s) = Hg(g) + Cl_2(g)$ (3)

Regarding HgO, direct evaporation can take place:

$HgO(s) = HgO(g)$ (4)

Alternatively, HgO can thermally decompose to Hg (ref[3]) followed by the evaporation of Hg:

$HgO(s) = Hg(g) + 1/2O_2(g)$ (5)

Furthermore, HgO may also undergo carbothermic reduction to Hg followed by the evaporation of Hg:

$HgO(s) + C(s) = Hg(l) + CO(g)$ (6)

For $HgSO_4$, direct evaporation can occur:

$HgSO_4(s) = HgSO_4(g)$ (7)

$HgSO_4$ can also thermally decompose to Hg (ref[4]) followed by the evaporation of Hg:

$HgSO_4(s) = Hg(g) + SO_2(g) + O_2(g)$ (8)

Based on our thermodynamic analysis (Supplementary Fig. 16a), the aforementioned decomposition and carbothermic reduction reactions are found to be spontaneous at temperature >1200 °C, which can be achieved using our HET process. Additionally, the vapor pressure of Hg and $HgCl_2$ is high at temperatures <500 °C (Supplementary Fig. 16b). Collectively, this analysis suggests that the HET process is viable for removal of Hg with different species.

Next, we used the HET process to remove Hg, HgO, and $HgSO_4$ from contaminated soil. These individual Hg species were added separately to the soil, which was then mixed with carbon black as conductive additives. The HET conditions remained consistent (100 V, 1 s) for all Hg contaminants (Supplementary Fig. 16c). Subsequently, we measured the removal efficiencies of each Hg species. As shown in Supplementary Fig. 16d, the HET process achieved high removal efficiencies for all Hg species: Hg (~90.4%), $HgCl_2$ (~94.6%), HgO (~95.1%), and $HgSO_4$ (~86.5%) using a single HET pulse of 1 s. It is worth noting that $HgCl_2$ possesses a higher vapor pressure compared to Hg (Supplementary Fig. 16b), leading to its slightly higher removal efficiency. Additionally, HgO is prone to decomposition into Hg (Supplementary Fig. 16a),

thereby exhibiting a high removal efficiency. On the other hand, the decomposition of $HgSO_4$ is relatively more challenging and occurs at higher temperatures (Supplementary Fig. 16a), resulting in a slightly lower removal efficiency compared to the other Hg species.

Supplementary Note 2. Strategy for scaling up the HET process.

To demonstrate the scalability of the HET process, we first conducted a theoretical analysis of the scaling rule of the HET process. Then, we performed batch-by-batch scaling up experiments in our research lab, with the productivity up to kg scale per day. Next, we proposed an ex-situ prototype for continuous HET processing using a belt roller. Finally, we provide a conceptual design of a tractor attached HET unit as well as a field test facility for in-site soil remediation.

2.1 Scaling rule of HET process by theoretical analysis.

Achieving effective removal of heavy metals and organic contaminants through the HET process largely depends on the maximum temperature reached. Therefore, ensuring consistent temperature across the sample is critical when scaling up the process. In Joule heating, the amount of heat (Q) can be calculated by Supplementary Equation 9,

$$Q = I^2Rt \quad (9)$$

where I is the current passing through the sample, R is the resistance of the sample, and t is the heating time. The amount of heat per unit volume (Q_v) can then be determined by Supplementary Equation 10,

$$Q_\mathrm{v} = j^2\rho_\mathrm{e}t \quad (10)$$

where j is the current density, ρ_e is the electrical resistivity of the sample, and t is the heating time.

The change in temperature (ΔT) is proportional to the amount of heat according to Supplementary Equation 11,

$$Q = C_{\mathrm{p}} m \Delta T \tag{11}$$

where C_{p} is heat capacity of the sample, and m is the mass of the sample. Supplementary Equation 6 can be revised to calculate heat per unit volume, as shown in Supplementary Equation 12,

$$Q_{\mathrm{v}} = C_{\mathrm{p}} \rho_{\mathrm{m}} \Delta T \tag{12}$$

where ρ_{m} is the density of the sample. For a specific kind of sample, maintaining a constant Q_{v} is crucial for consistent temperature control as C_{p} and ρ_{m} are constant.

Since the electrical resistivity (ρ_{e}) of the sample is constant, to maintain a constant Q_{v} and t while scaling up the sample, a constant j must be maintained, as per Supplementary Equation 10.

The charge (q) in the capacitor bank can be calculated by Supplementary Equation 13,

$$q = CV \tag{13}$$

where C is the total capacitance, and V is the charging voltage. Assuming the charges in the capacitor bank are discharged in the heating time (t), the current (I) passing through the sample can be calculated by Supplementary Equation 14,

$$I = \frac{q}{t} \tag{14}$$

Hence, the current density (j) can be determined by Supplementary Equation 15,

$$j = \frac{I}{S} = \frac{CV}{St} \tag{15}$$

where S is the sample cross-sectional area. Since the samples are cylinder-shaped in a quartz tube, the sample mass (m) can be calculated by Supplementary Equation 16,

$$m = \rho_{\mathrm{m}} S L \tag{16}$$

where ρ_{m} is the sample density, S is the sample cross-sectional area, and L is the sample length. ρ_{m} is constant considering the same compression of the sample.

We can further obtain Supplementary Equation 17 to determine the current density,

$$j = \frac{CV\rho_{\mathrm{m}}L}{mt} \quad (17)$$

As discussed earlier, maintaining a constant current density (j) is necessary to increase the sample mass (m), which can be achieved through practices including: (1) linearly increasing the HET voltage (V), and (2) linearly increasing the capacitance (C).

2.2 Demonstration of the scaling of the HET process in our research lab.

In our first-generation HET system, the capacitor bank is made up of 10 commercial aluminum electrolytic capacitors (450 V, 6 mF, Mouser #80-PEH200YX460BQU2) with a total capacitance of C_0 = 0.06 F. In our small-scale experiment (Supplementary Table 2), we used a sample mass of m_0 = 0.2 g and HET conditions of V_0 = 100 V and C_0 = 0.06 F, resulting in a temperature of ~3000 °C (Fig. 1d). Here, we have successfully scaled up the HET to a larger sample mass of m_1 = 2 g (Supplementary Fig. 29). For this purpose, we built a second-generation HET system (Supplementary Fig. 29a) with a larger capacitor bank and the total capacitance reaches C_1 = 0.624 F. According to Supplementary Equation 10, we derived the formula in Supplementary Equation 18,

$$\frac{m_1}{m_0} = \frac{C_1V_1}{C_0V_0} \quad (18)$$

For the sample mass of m_1 = 2 g and C_1 = 0.624 F (Supplementary Fig. 29b), we used a HET voltage of V_1 = 120 V, which fit the Supplementary Equation 18. Since the achievable temperature is critical for the heavy metal removal by evaporation and organic contaminants removal by graphitization, the temperature for the large-scale sample was recorded (Supplementary Fig. 29c). The maximum temperature also reached ~3000 °C, demonstrating the successful scaling up of the HET process. The heavy metal concentration in the r-Soil was reduced, and the removal efficiency

of heavy metals was calculated to be 40 to 80% for one-time HET (Supplementary Fig. 29d), comparable to that of the small-scale sample (Fig. 2c).

In addition, we have further upscaled the sample mass to ~8 g per batch using Metcoke as the conductive additive (Supplementary Fig. 29e), and achieved a total treated soil mass of ~100 g with processing time of <10 min after sieving separation of the Metcoke (Supplementary Fig. 29f). As a result, we are currently able to achieve a production rate of >10 kg day^{-1} in our research lab.

2.3 Use of the AC-HET process for scaling up to 100 g per batch.

We integrated an alternating current (AC) supply with the HET process, which offers better scalability compared to the direct current (DC) process (Supplementary Fig. 30). We have extended the AC supply to treat a sample mass of 100 g per batch (Supplementary Fig. 31). Pyrene-contaminated soil was used as a representative. The soil was mixed with Metcoke as the conductive additive and loaded into a quartz tube with an inner diameter of 4 cm (Supplementary Fig. 31a). The sample was connected to the AC system for thermal treatment (Supplementary Fig. 31b). After treatment, the mixture of treated soil and Metcoke was obtained (Supplementary Fig. 31c), and the Metcoke was removed by sieving, resulting in separated treated soil (Supplementary Fig. 31d). After two cycles of AC-HET treatment, the pyrene concentration in the soil was reduced to below the safe content (Supplementary Fig. 31e-f), demonstrating the effectiveness of the HET process for the removal of polycyclic aromatic hydrocarbons (PAHs). The treatment process took approximately 1 minute for the 100 g soil treatment. This production rate corresponds to ~6 kg h^{-1} or ~144 kg day^{-1}.

2.4 The prototypes of continuous HET using a belt roller.

The HET process can be integrated with various industrial scale-up technologies. Here, we introduce a prototype assembly of the HET process with a belt roller for continuous processing (Supplementary Fig. 32a). In this assembly, the c-Soil/CB mixture is loaded into a chamber, compressed to proper resistance, undergoes the HET process, and finally, the remediated soil is unloaded. We note that this is just one possible method for continuous processing, and other established industrial scaling techniques could also be applied.

In another design (Supplementary Fig. 32b), the HET process can be conducted in a continuous manner. The thin and flexible sheet metal belt is used for soil conversion. As the sheet metal is not a good electrical conductor along its length, the current is concentrated where the electrodes are located, i.e., the flash zone. The top electrode is segmented longitudinally, consisting of a stack of plates with thin insulation separating them. If there is non-uniform resistance in the sample, the current may become localized, resulting in parts of the sample intensely heated, and other higher resistance parts minimally heated. In this scheme, the current in each wire to each segment will be the same, as each will have a separate current control, such as an insulated-gate bipolar transistor (IGBT) or zero-crossing relay if AC power is used. A resistor divider circuit is a low-cost option but less energy-efficient. Temperature sensors can be embedded in the tip of each electrode segment to assure that all parts of the moving sample are heated equally. The wire leads are shown separately for visualization, but would all be in a row perpendicular to the plane of the drawing plane. This allows for an indefinite width of the belts by adding more electrode segments.

2.5 Conceptual design of a tractor attached HET unit for on-site soil remediation.

Considering the high cost of excavating and transporting contaminated soil to a remediation facility, as well as the difficulty in providing electricity to remote areas, we here

propose a conceptual design for on-site soil remediation using a tractor-attached HET unit (Supplementary Fig. 33). In this design, the HET is powered by a diesel generator or rechargeable batteries. The process involves the following steps: (1) Disc ploughing the soil to soften it. (2) Excavating and converting the contaminated soil using a sheet metal belt and tensioning roller system. (3) Drying the soil to reduce moisture content, if necessary. (4) Adding carbon additives and mixing them with the dried soil. (5) Compressing the mixture to an appropriate resistance. (6) Joule heating the mixture using electricity provided by the generator and collecting the heavy metal volatiles in a trap. (7) Separating the carbon additives from the soil by sieving. (8) Redepositing the remediated soil at nearly its original location.

In addition, the HET process can be integrated with traditional thermal desorption method for soil remediation. We also propose a modified facility design based on a known method[5], but in our case the electrodes provide a rapid voltage pulse for electric heating rather than long-duration heat injection (Supplementary Fig. 34). The facility includes a vacuum well, vacuum piping, collector, filtration, blower, and exhaust stack. In this design, the contaminated soil is considered as a dry, porous material. Soil is firstly mixed with carbon conductive additives. The electrodes are installed in the heating well, with the depth determined by the level of contamination. A vacuum collection system is designed to capture the volatile matter during the HET process.

2.6 Consideration of the soil moisture.

We have also tested the applicability of the HET process for remediation of moisture-containing soil. We measured the moisture content of the soil used in our experiments. The previously used dry soil had a moisture content of ~1.2% (Supplementary Fig. 35a). We collected another batch of soil with a moisture content of ~14% (denoted as Moisture soil, Supplementary Fig. 35b). Pyrene,

a representative contaminant, was added to the moisture soil, which was then mixed with carbon black as the conductive additive. The electric input of the HET treatment was the same as that for the dry soil (Supplementary Table 2). The intensity of pyrene absorption peaks progressively decreased with increasing electric pulses (Supplementary Figs. 35c-d), similar to the results obtained from the dry soil (Figs. 3a-b). The pyrene removal efficiency in the moisture soil reaches 91% after 3 HET pulses, which is slightly lower than that of the dry soil (95%). This demonstrates the feasibility of the HET process for remediation of moisture-containing soil.

Supplementary Note 3. The electrical energy cost evaluation of the HET process.

The energy consumption is calculated using Supplementary Equation 19,

$$E = \frac{(V_1^2 - V_2^2) \times C}{2 \times M} \quad (19)$$

Where E is the energy per gram (kJ g^{-1}), V_1 and V_2 are the capacitor voltages before and after HET, respectively, C is the capacitance (60 mF), and M is the mass per batch.

For a typical trial with V_1 = 100 V, V_2 = 0 V, and M = 0.2 g, the energy consumption was calculated to be,

E = 1.50 kJ g^{-1} = 4.20 × 10^{-4} kWh g^{-1} = 420 kWh $tonne^{-1}$

For the second-generation system with C_2 = 0.624 F (Supplementary Note 2, Supplementary Fig. 29), and using the upscaled sample parameters V_1 = 200 V, V_2 = 0 V, and M = 8 g, the energy consumption was calculated to be,

E = 1.56 kJ g^{-1} = 4.37 × 10^{-4} kWh g^{-1} = 437 kWh $tonne^{-1}$

This value is consistent with the small-scale sample. Theoretically, the electric energy density plays a crucial role in the HET remediation process. As long as the energy density remains the same during the scaling-up process, the estimation of energy consumption will remain consistent.

We conducted a cost comparison between the HET purification process and established thermal remediation methods, which use heat to eliminate contaminants through thermal desorption, destruction, or immobilization. Traditional thermal remediation techniques include thermal conduction heating (TCH), steam-enhanced extraction (SEE), electrical resistance heating (ERH), and radio frequency heating (RFH)[6]. The energy consumption of the HET process is comparable to or lower than these traditional methods (Supplementary Table 3, Supplementary Fig. 36), despite the fact that the HET process allows for a much higher temperature and thus can remediate a wider range of contaminants with less volatility. Furthermore, even though the HET process operates at a higher temperature than traditional thermal methods, its processing time is significantly shorter, resulting in similar or lower energy consumption. In addition, the HET process is more energy-efficient than other innovative electricity-based remediation techniques such as the electrochemical process[7].

Supplementary Note 4. Life-cycle assessment and techno-economic analysis

4.1 Goal and scope

This study is conducted in accordance with the requirements outlined in ISO 14044 (ref[8]).The primary objective is to assess the environmental and energy impact, as well as the cost, of the HET process for soil remediation. Specifically, the analysis aims to determine whether the newly established HET process reduces energy consumption and water consumption compared to

other soil remediation methods[9] such as thermal desorption[10], soil washing[11], and chemical degradation[12]. Additionally, the study evaluates the cost competitiveness of the HET process in comparison to these established soil remediation techniques.

4.2 Scenario description and system boundaries

In this study, four scenarios were examined (Supplementary Fig. 37, Supplementary Table 4), with each scenario representing a different soil remediation method. The functional unit used in all scenarios is the off-site remediation of 1 tonne of PAH-contaminated soil, and all other materials flow are normalized accordingly. It is important to note that the transportation and pretreatment steps such as pre-drying are not considered in this analysis.

Scenario 1 HET. In this scenario, 1 tonne of contaminated soil is mixed with 0.5 tonne of conductive carbon (Metcoke in this study). The soil/carbon mixture is then treated using the HET process. The resulting treated soil/carbon mixer weighs 1.335 tonnes, which is then separated by sieving. A recovery yield of 92% is assumed for Metcoke recycling (Supplementary Fig. 10), resulting in a Metcoke consumption of 0.04 tonne.

Scenario 2 Thermal Desorption. In this scenario, a typical thermal desorption process is employed[13], where 1 tonne of contaminated soil is treated at 350 °C for 90 min using a furnace.

Scenario 3 Soil Washing. In this scenario[11], 1 tonne of contaminated soil is extracted with a 4000 L of mixture solvent (5% pentanol-10% water-85% ethanol) using a rotating shaker at 16 rpm for 1 h. The treated soil/solvent is then separated by filtration. The soil washing process is conducted for 3 cycles, consuming 600 L of 1-pentanol (0.488 tonnes), 1200 L of water (1.2 tonnes), and 10200 L of ethanol (8.048 tonnes). It is assumed that the solvents can be recovered, with a recovery yield of 98%, although the specific recovery process is not accessed in this study.

Scenario 4 Chemical Oxidation. In this scenario[12], 1 tonne of contaminated soil is mixed with a mixture solution containing 3.22 tonnes of H_2O_2 (30%) and 85.5 tonnes of H_2O. Then, the mixture is agitated at 200 rpm for one week. Finally, the soil and wastewater are separated by filtration. It is assumed that the chemicals used in this process can be recovered, with a recover yield of 90%, although the recovery process is not accessed in this study.

4.3 Life-cycle inventory

The water consumption and energy consumption values for raw materials and processing are summarized in Supplementary Table 5, which are explained in detail below.

Raw materials. The values for coke from coal production for steel manufacturing, H_2O_2 (30%), and ethanol production from corn-wet milling corn ethanol are obtained from US department of energy (DOE) GREET Model[14]. Ethanol is used as a proxy for 1-pentanol since 1-pentanol is not included in the GREET databases.

Processing – Mixing. Energy input is required for the mixing of conductive carbon with contaminated soil. It is assumed that the mixing is conducted using an electricity-driven Powder Mixer[15], with an estimated energy consumption of 9.432 MJ $tonne^{-1}$. No water consumption is involved in this process.

Processing – HET. Energy consumption for the HET process is estimated to be 1512 MJ $tonne^{-1}$ (Supplementary Note 3). No water consumption in associated with this process.

Processing – sieving. The separation process is assumed to be conducted using an industrial vibrating sieving system. For an electrical shaker machine, the estimated energy consumption is ~4 MJ $tonne^{-1}$. No water consumption occurs during the sieving process.

Processing – Furnace heating. In the case of thermal desorption, it is assumed that furnace heating is carried out using an electrical furnace[16], with a load of 45 kg, power of 10 kWh, and heat-up time of 2 h. The treatment time is 90 min. The energy consumption is estimated to be ~778 kWh tonne^{-1}, or 2800 MJ tonne^{-1}. This value is comparable to the reported energy consumption values for thermal desorption found in literatures (Supplementary Fig. 36, Supplementary Table 3).

Processing – Agitation. It is assumed that an industrial agitator[17] with a power of 0.4 kW, tank capacity of 0.5 m^3, and operating time of 1 h, is used. The energy consumption is calculated to be 0.8 kWh tonne^{-1}, or 2.88 MJ tonne^{-1}.

Processing – Filtration. The filtration process is assumed to be conducted using a filtration system. The energy consumption is estimated to be ~2.2 MJ tonne^{-1}.

4.4 Life-cycle impact assessment

In this study, the environmental impacts are assessed using two indicators, cumulative water use (Supplementary Table 6) and cumulative energy demand (Supplementary Table 7).

4.5 Cost evaluation

In this study, the cost of raw materials is based on the prices of commercial products, including metallurgical coke ($150 tonne^{-1}, ref[18]), water ($0.5 tonne^{-1}, ref[19]), 30% H_2O_2 ($350 tonne^{-1}, ref[20]), 1-pentanol ($1000 tonne^{-1}, ref[21]), and ethanol ($800 tonne^{-1}, ref[22]). The cost of energy consumption is calculated using the industrial electricity rate in Texas, US ($0.0587 KWh^{-1}, US Energy Information Administration, ref[23]). These values are listed in Supplementary Table 8. The materials expense and energy expense in electricity are calculated and summarized in Supplementary Table 9. The Operating Expense is then calculated as the sum of the materials

expense and energy expense. It is important to note that labor costs are not included in the Operating Expense.

Next, we estimated the Capital Expense. For the HET process, the Equipment Price of the homemade lab-scale HET equipment (Supplementary Fig. 29) is ~$5000, with an Annual Production of ~30 tonnes (assuming 10 g per batch with a treatment duration of 10 s). The Capital Expense is calculated by:

$$Capital\ Expense\ = \frac{Equipment\ Price}{Useful\ Lifetime} \times \frac{1}{Annual\ Production} \qquad \text{(S20)}$$

Assume a Useful Lifetime ranging from 10 to 20 years, the Capital Expense is calculated to be $8.35 to $16.7 tonne^{-1}. The Total Expense is then calculated by:

$$Total\ Expense = Capital\ Expense + Operating\ Expense \qquad \text{(S21)}$$

The Total Expense of the HET process ranges from $51.7 to $60 tonne^{-1}.

Comparatively, the typical Total Expense of ex-situ thermal desorption ranges from $46 to $99 per tonne (ref[24]). The typical Total Expense of soil washing ranges from $50 to $165 per tonne (ref[25]). For chemical oxidation, the main equipment used is an agitator[12], with a typical cost of $1800 and a loading capacity of 10000 L, equivalent to ~10 tonnes (ref[26]). Assuming an Annual Production being 10 tonne of contaminated soil and a Useful Lifetime of 10 to 20 years, the Capital Expense is calculated to be $9 to $18 per tonne of contaminated soil. The total Expense of the chemical oxidation process is estimated to be $172 to $181 per tonne of contaminated soil.

4.6 Sensitivity and Uncertainty.

There are several important considerations and potential sources of uncertainty in this study. Firstly, the water consumption, energy consumption, and price data for raw materials used in this study were obtained from different sources, which may introduce some uncertainty.

Secondly, several assumptions were made in this study regarding the recovery of materials and the processing parameters, such as mixing, sieving, agitation, and filtration. These assumptions can contribute to uncertainty in the findings. Thirdly, although we propose the potential scalability of the HET process for soil remediation, it is important to note that the experiments and data presented in this work are conducted at a small scale (in kg). As a result, there will be inherent uncertainty when scaling up the HET process to a larger tonne-scale application, including factors such as labor expense, capital expense, and operating expense. Therefore, it is crucial to consider these uncertainties and limitations when interpreting the results, and further research and validation at larger scales will be necessary to provide more accurate assessments of energy consumption, cost, and overall feasibility of the HET process for commercial-scale soil remediation.

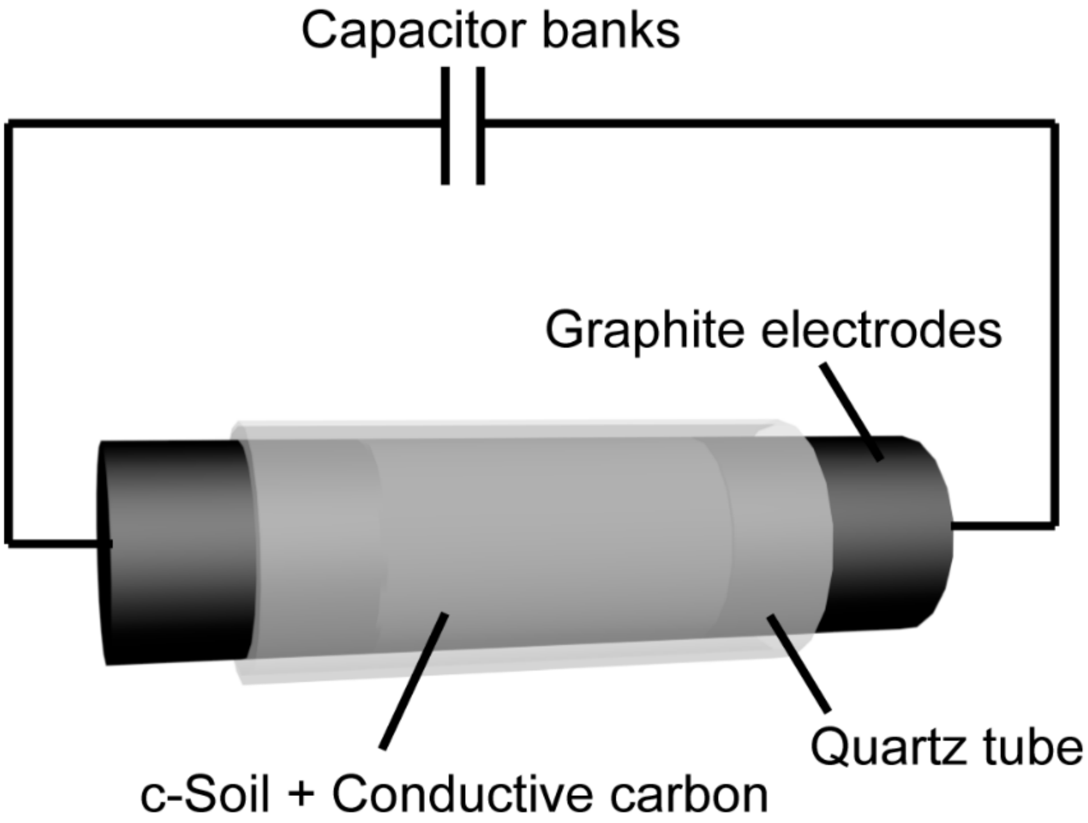

Supplementary Fig. 1. Scheme of the bench-scale setup of the HET process. c-Soil, contaminated soil.

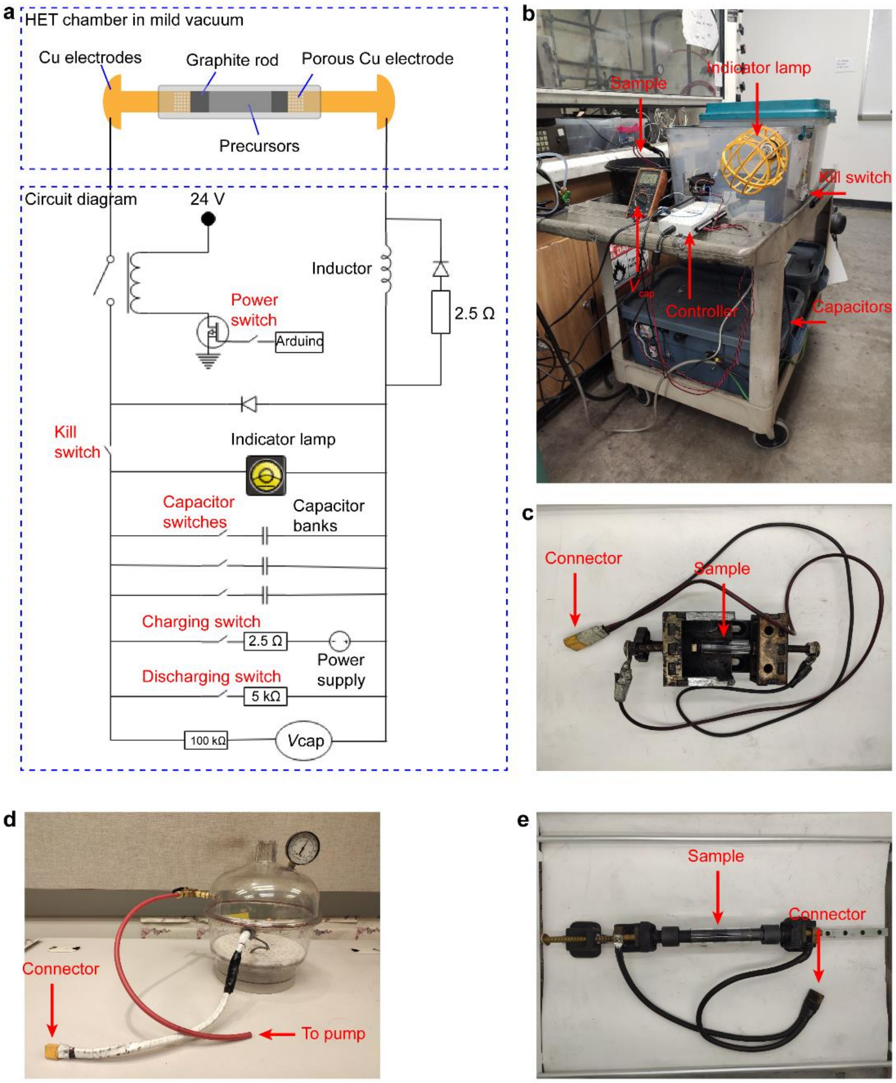

Supplementary Fig. 2. Electrical diagram and setup of the high-temperature electrothermal (HET) system. (**a**) Electrical diagram of the system. The capacitor bank is composed of 10 aluminum capacitors (450 V, 6 mF, Mouser #80-PEH200YX460BQU2). The total capacitance is 60 mF. The detailed description of the electrical components could be found in our previous publication[27]. The graphite electrodes are loosely loaded in the quartz tube, and porous Cu electrodes are used to permit outgassing. (**b**) Picture of the HET system. (**c**) Picture of the reaction stage for small samples. (**d**) Picture of the mild vacuum chamber. (**e**) Picture of the reaction stage for large samples.

CAUTION: There is a risk of electrical shock if improperly operated. We recommended the below safety guidance when using this equipment. More safety practices could be found in our previous publications[27,28].

1. Enclose or carefully insulate all wire connections.
2. All connections, wires, and components must be suitable for the high voltages and currents.
3. One hand rule. Use only one hand when working on the system, with the other hand not touching any grounded surface.
4. Provide a mechanical discharge circuit breaker switch connected to a power resistor of a few hundred ohms to rapidly bleed off the capacitor charge.
5. Provide a “kill” circuit breaker switches to disconnect the sample holder from the capacitor bank.
6. Post high voltage warning signs on the apparatus.
7. Keep in mind that the system can discharge many thousands of Joules in milliseconds, which can cause components such as relays to explore.
8. Keep a voltmeter with high voltage test leads handy at all times. When working on the capacitor bank, always check the voltage on each.
9. Wear thick rubber gloves when using the apparatus to protect from electrocution.
10. Safety glasses for welding are recommended to block the infrared and ultraviolet light during the flashing reaction.
11. The reliability and robustness of the HET system should be confirmed by an experienced electrical technician with weekly re-inspections.
12. All users should be properly trained by an experienced electrical technician.

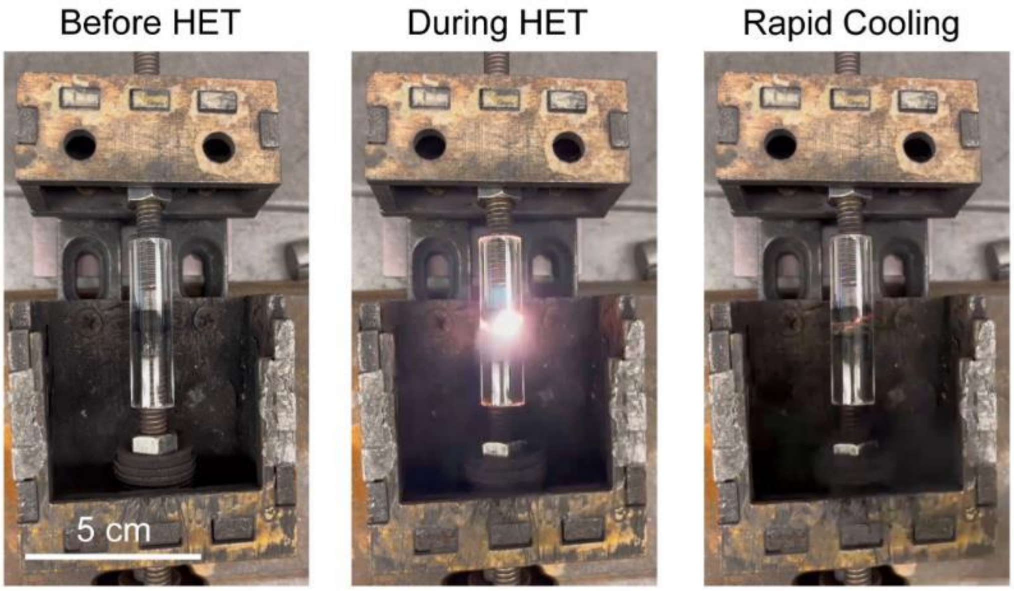

Supplementary Fig. 3. Picture of the sample during high-temperature electrothermal process (HET). The rapid cooling is enabled by thermal radiation.

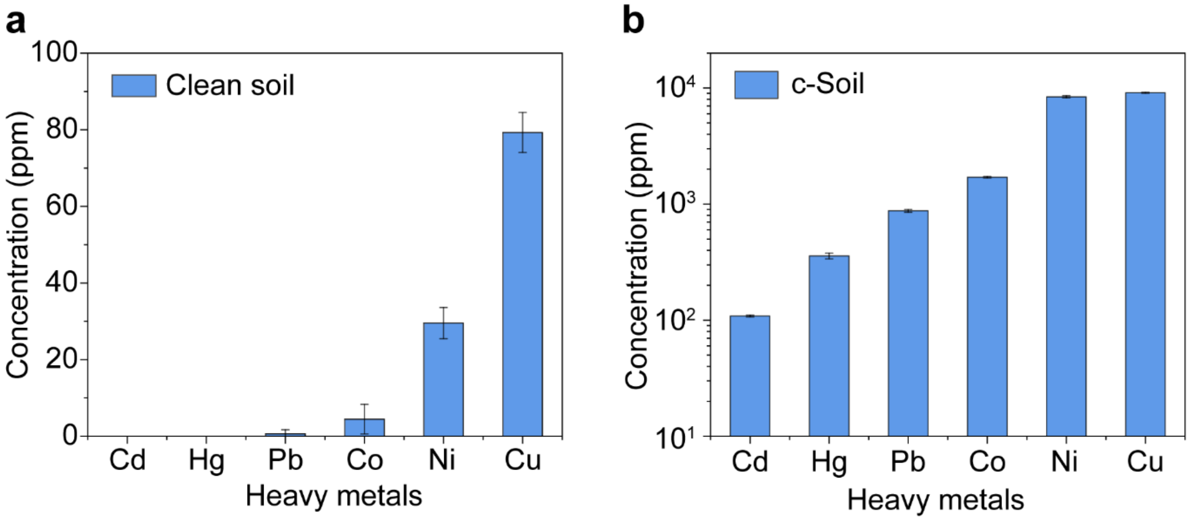

Supplementary Fig. 4. Heavy metal contents in as-collected clean soil and contaminated soil. (**a**) The heavy metal contents in clean soil. (**b**) The heavy metals contents in contaminated soil (c-Soil). The concentration of 0 denotes undetectable content by ICP-OES. The error bars denote SD where N = 3.

The concentrations of heavy metals in clean soil are low (Cd undetectable, Hg undetectable, Pb ~0.6 ppm, Co ~4.5 ppm, Ni ~30 ppm, and Cu ~79 ppm), therefore the concentration of heavy metals in the contaminated soil are controlled by spiking with metal salts (Cd ~100 ppm, Hg ~300 ppm, Pb ~1000 ppm, Co ~2000 ppm, Ni ~10000 ppm, and Cu ~10000 ppm).

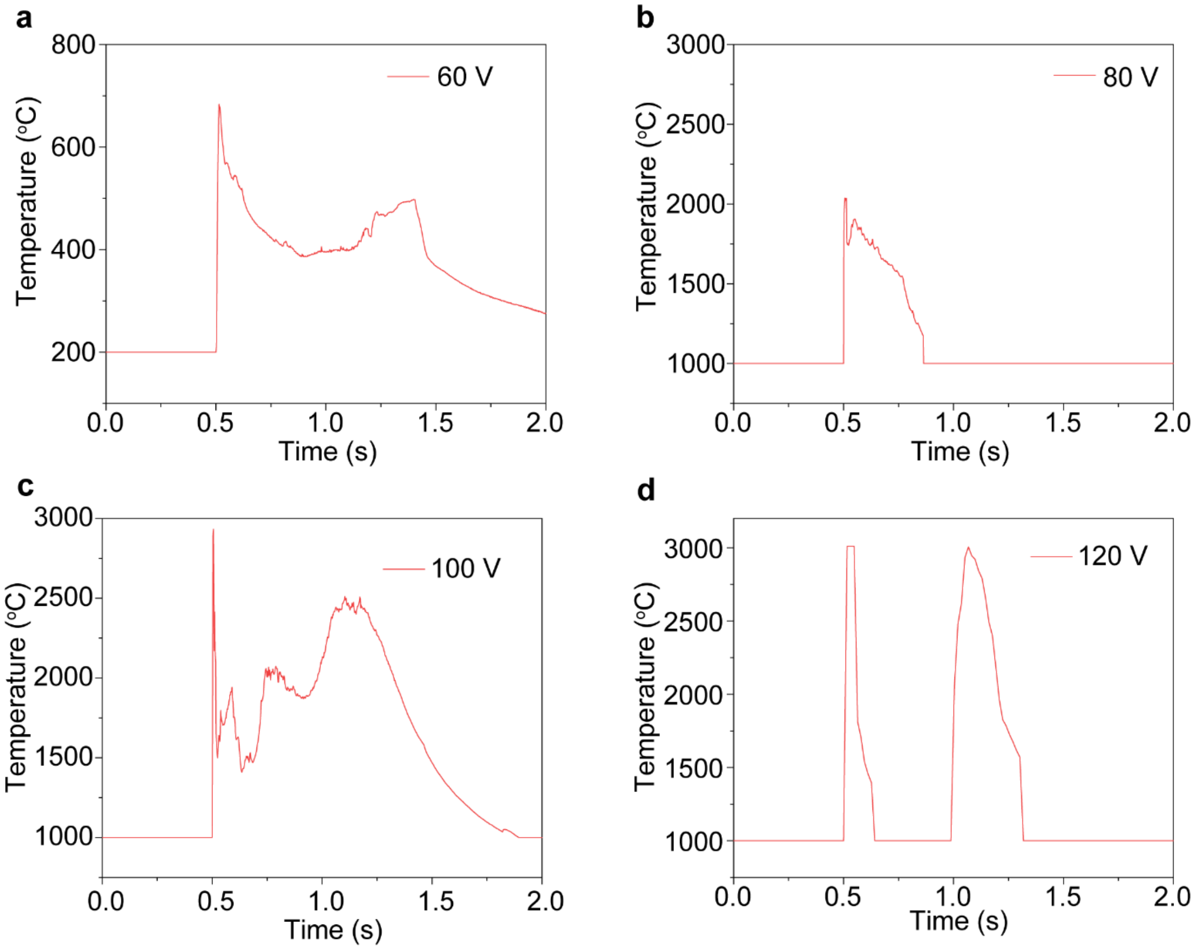

Supplementary Fig. 5. Temperature measurement under different voltage input. Temperature profile of HET at voltages of (**a**) 60V, (**b**) 80 V, (**c**) 100 V, and (**d**) 120 V.

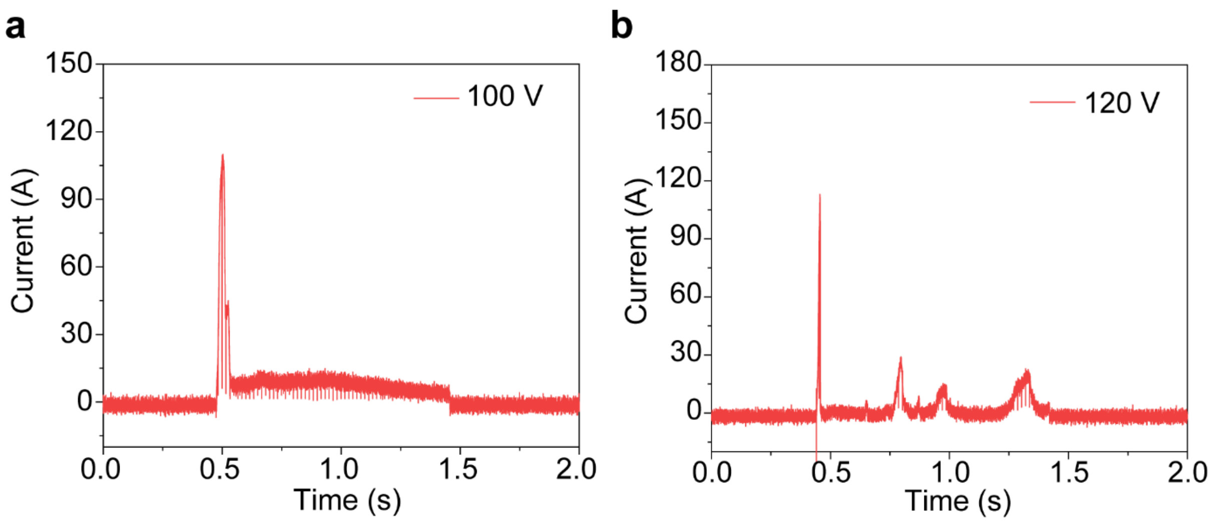

Supplementary Fig. 6. Current measurement. (**a**) Current profile under HET voltage of 100 V. (**b**) Current profile under HET voltage of 120 V.

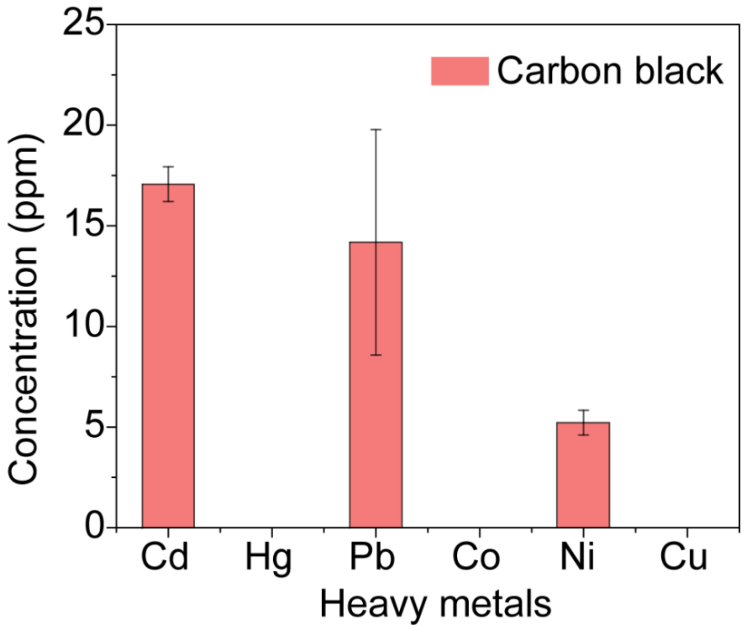

Supplementary Fig. 7. Heavy metal contents in carbon black. The heavy metals contents in carbon black. The error bars denote the SD where N = 3. The concentration of 0 denotes undetectable content by ICP-OES.

The concentrations of heavy metals in carbon black (Cd ~17 ppm, Hg undetectable, Pb ~10 ppm, Co undetectable, Ni ~6 ppm, and Cu undetectable) are far below that of the contaminated soil (Cd ~100 ppm, Hg ~300 ppm, Pb ~1000 ppm, Co ~2000 ppm, Ni ~10000 ppm, and Cu ~10000 ppm), and hence will not introduce significant error during the HET process.

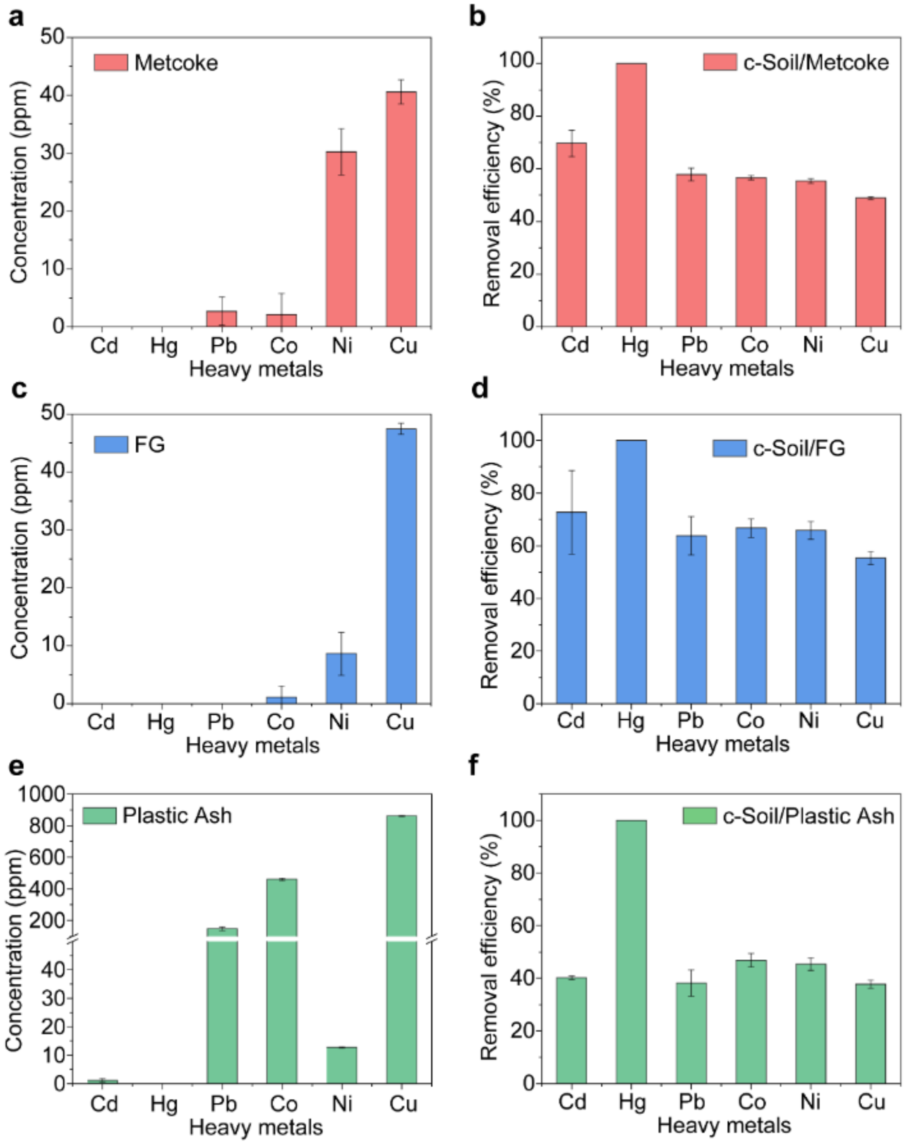

Supplementary Fig. 8. Heavy metals removal using varied inexpensive carbon source additives. (**a**) Heavy metal contents in metallurgical coke (Metcoke). (**b**) Heavy metal removal efficiencies using Metcoke as the conductive additives. (**c**) Heavy metal contents in flash graphene

(FG) derived from Metcoke. (**d**) Heavy metal removal efficiencies using FG as the conductive additives. (**e**) Heavy metal contents in plastic pyrolysis ash (Plastic Ash). (**f**) Heavy metal removal efficiencies using Plastic Ash as the conductive additives. The error bars in all plots denote the SD where N = 3.

The heavy metals concentrations in Metcoke are as below: Cd undetectable, Hg undetectable, Pb ~2.7 ppm, Co ~2.1 ppm, Ni ~30 ppm, and Cu ~41 ppm. These values are significantly lower than those in the contaminated soil (Cd ~100 ppm, Hg ~200 ppm, Pb ~1000 ppm, Co ~200 ppm, Ni ~10000 ppm, and Cu ~10000 ppm), and hence the use of Metcoke as conductive additive during the HET process will not introduce significant error. The removal efficiencies of heavy metals with Metcoke as an additive are ~60% for most of the metals, slightly lower than those with carbon black as an additive (Fig. 2c). The difference may be due to the higher conductivity of carbon black compared to Metcoke (R ~1.0 Ω for CB as an additive, and R ~2.0 Ω for Metcoke as an additive), resulting in better removal efficiencies. Additionally, the smaller particle size and higher surface area of carbon black could result in more homogeneous heating, providing better removal efficiencies than Metcoke.

Flash graphene (FG) was also used as a conductive additive . The FG is synthesized using Metcoke as the precursor. The concentrations of heavy metals in the FG are as below: Cd undetectable, Hg undetectable, Pb undetectable, Co ~1.1 ppm, Ni ~8.6 ppm, and Cu ~47 ppm. These values are slightly lower than those in the raw materials due to evaporative loss of heavy metals during the FG synthesis. In addition, these values are well below the contaminated soil levels (Cd ~100 ppm, Hg ~200 ppm, Pb ~1000 ppm, Co ~200 ppm, Ni ~10000 ppm, and Cu ~10000 ppm), posing no significant error during the HET process. The removal efficiencies of heavy metals are >60%, which is slightly less than that of carbon black as an additive (Fig. 2c).

The reason might be similar with that of the Metcoke as an additive: firstly, carbon black has superior conductivity to FG (R ~1.0 Ω for CB as an additive, and R ~1.5 Ω for FG as an additive); Secondly, carbon black has a much smaller particle size and much higher surface area than FG.

Furthermore, plastic pyrolysis ash (Plastic Ash) was tested as a conductive additive. The concentrations of heavy metals in the Plastic Ash are as below: Cd ~1.1 ppm, Hg undetectable, Pb ~148 ppm, Co ~461 ppm, Ni ~12 ppm, and Cu ~862 ppm. These values are notably higher than those found in CB, Metcoke, or FG. Most heavy metal concentrations in the Plastic Ash are below those of the contaminated soil (Cd ~100 ppm, Hg ~200 ppm, Pb ~1000 ppm, Co ~200 ppm, Ni ~10000 ppm, and Cu ~10000 ppm), except for Co, which has a higher content in Plastic Ash than the contaminated soil. Thus, when Plastic Ash was used as a conductive additive, the concentration of heavy metals in the Plastic Ash must be taken into consideration. To evaluate the removal efficiency in this case, the following equation was used,

$$R = \frac{c(\text{c−Soil})\times m(\text{c−Soil})+c(\text{Plastic Ash})\times m(\text{Plastic Ash}) - c(\text{r−Soil})\times m(\text{r−Soil})}{c(\text{c−Soil})\times m(\text{c−Soil})+c(\text{Plastic Ash})\times m(\text{Plastic Ash})} \times 100\% \qquad (22)$$

where the mass of c-Soil used for HET was m(c-Soil), the concentration of heavy metals in c-Soil was measured as c(c-Soil), the mass of Plastic Ash used for HET was m(Plastic Ash), the concentration of heavy metals in Plastic Ash was measured as c(Plastic Ash), the mass of the remaining solid (mixture of r-Soil and residual graphene) after HET was m(r-Soil), and the concentration of heavy metals in the remaining solid was measured as c(r-Soil). The removal efficiencies of heavy metals are >40%, which is slightly lower than that achieved by using carbon black as an additive (Fig. 2c). This may be due to similar reasons as with the Metcoke as a conductive additive: firstly, carbon black has better conductivity than Plastic Ash (R ~1.0 Ω for carbon black as an additive, and R ~3.0 Ω for Plastic Ash as an additive); secondly, carbon black has a smaller particle size and a higher surface area than Plastic Ash.

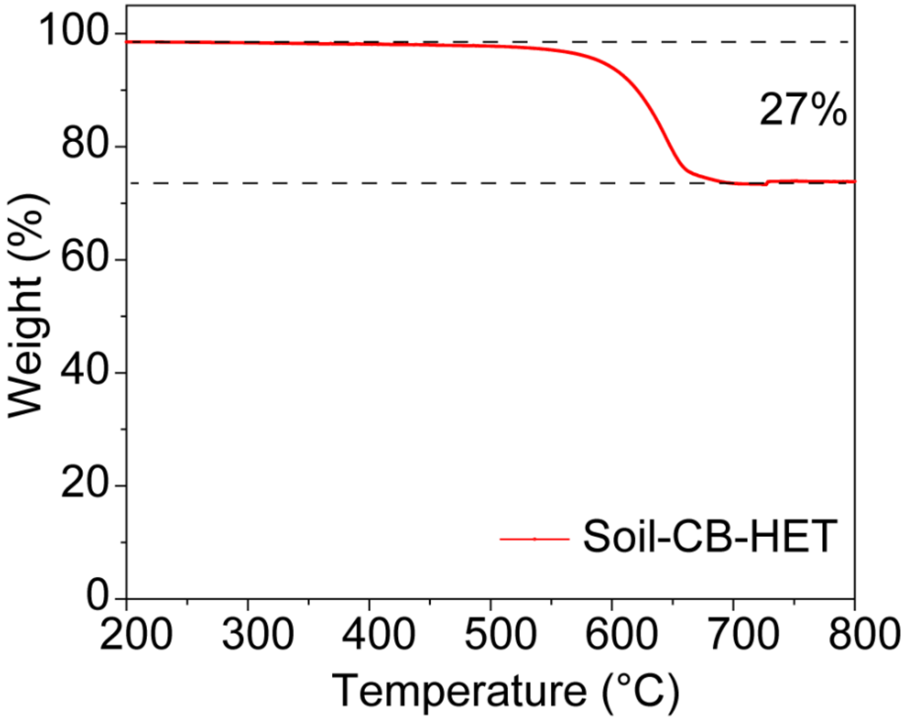

Supplementary Fig. 9. Carbon residue in the r-Soil. TGA curve of the r-Soil with residual carbon. TGA was conducted in air with the heating rate of 10 °C min^{-1}.

During the HET process, the easy-to-decompose components in the soil were decomposed. Hence, in the TGA measurement, most of the weight loss was ascribed to the residual carbon.

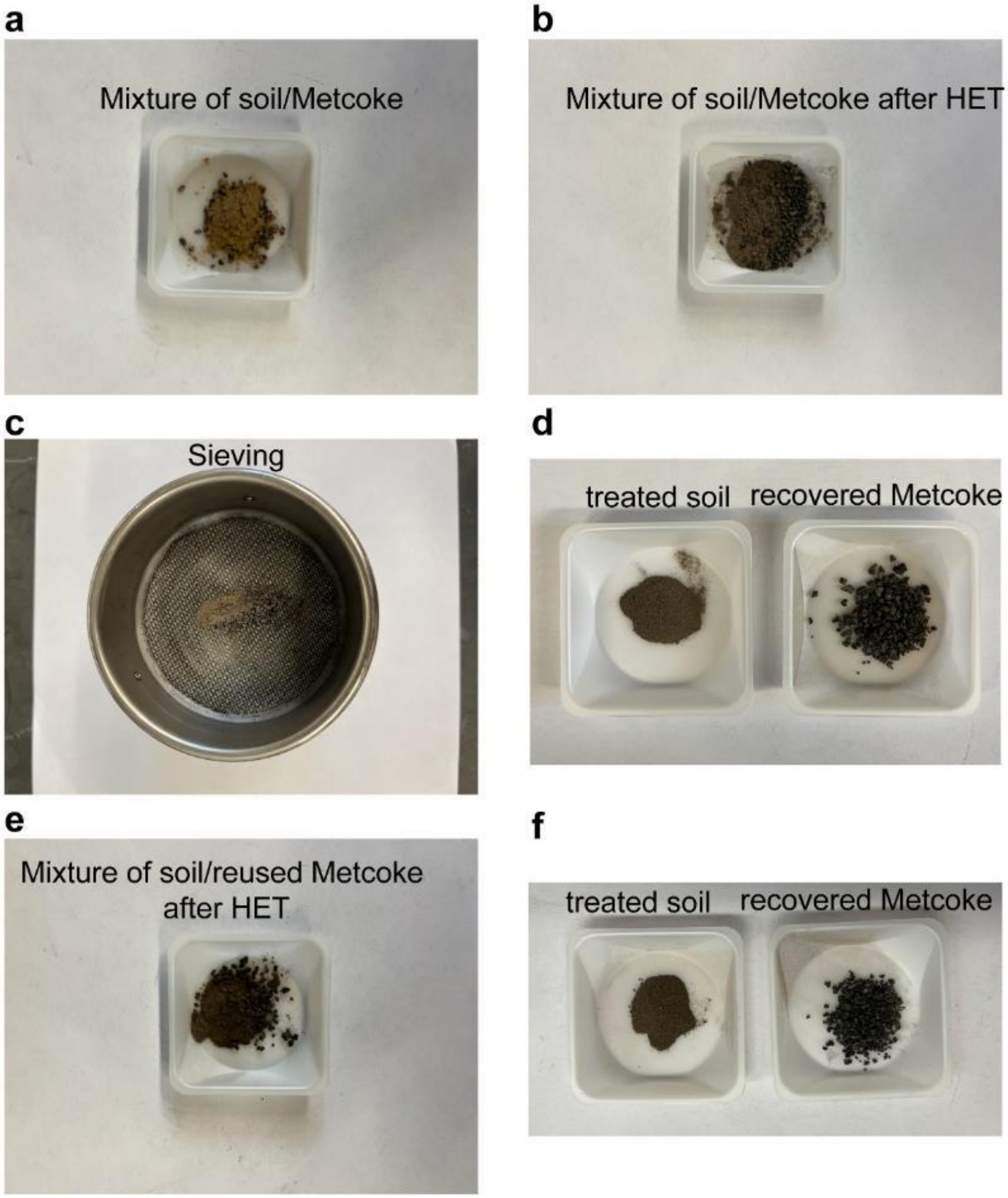

Supplementary Fig. 10. Separation and reuse of the conductive additive, metallurgical coke (Metcoke). (**a**) Picture of the mixture of soil/Metcoke before high-temperature electrothermal process (HET). (**b**) Picture of the mixture of soil/Metcoke after HET. (**c**) Separation of soil and Metcoke by sieving. (**d**) Picture of the separated treated soil and recovered Metcoke. (**e**) Picture of the mixture of soil/reused Metcoke after HET. The Metcoke is mostly recovered from previous HET trail. (**f**) Picture of the separated treated soil and recovered Metcoke.

Based on the density and particle size difference between soil and the conductive additives, we were able to separate them by physical processes. By using metallurgical coke (Metcoke) as an example, we demonstrated the separation and reuse of the conductive additive. We selected Metcoke with a particle size larger than that of soil (Supplementary Fig. 10a). After the HET purification process, the particle size of the Metcoke remained larger than that of the soil (Supplementary Fig. 10b). Hence, we were able to separate the soil and Metcoke by sieving (Supplementary Figs. 10c-d). In a typical experiment, we used a mixture of soil and Metcoke with a mass of m(soil) = 334 mg and m(Metcoke) = 166 mg. After HET, the remaining mass of soil and Metcoke was m(soil+Metcoke, HET) = 445 mg. After sieving separation, we obtained purified soil with a mass of m(purified soil) = 293 mg and recovered Metcoke with a mass of m(recovered Metcoke) = 152 mg, resulting in a recovery yield of Metcoke of ~92%. The ~8% mass loss might be attributed to consumption during the HET process.

We then used the recovered Metcoke (152 mg) and some fresh Metcoke (14 mg) as the conductive additive for a second HET (Supplementary Fig. 10e). After a similar sieving separation, we recovered Metcoke with a mass of m(recovered Metcoke) = 155 mg (Supplementary Fig. 10f), resulting in a recovery yield of Metcoke of ~93%. This demonstrated that simple sieving recycling could significantly reduce the consumption of conductive additive.

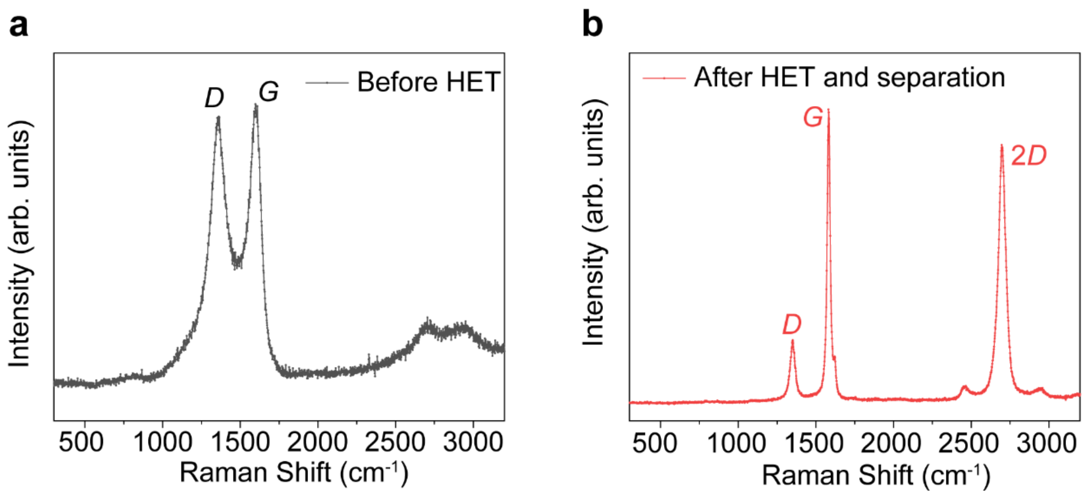

Supplementary Fig. 11. Raman spectrum of Metcoke. (**a**) Raman spectrum of the Metcoke raw materials. (**b**) Raman spectrum of Metcoke after high-temperature electrothermal process (HET) and separation.

The Raman spectra showed that the Metcoke was transferred to flash graphene after the HET process.

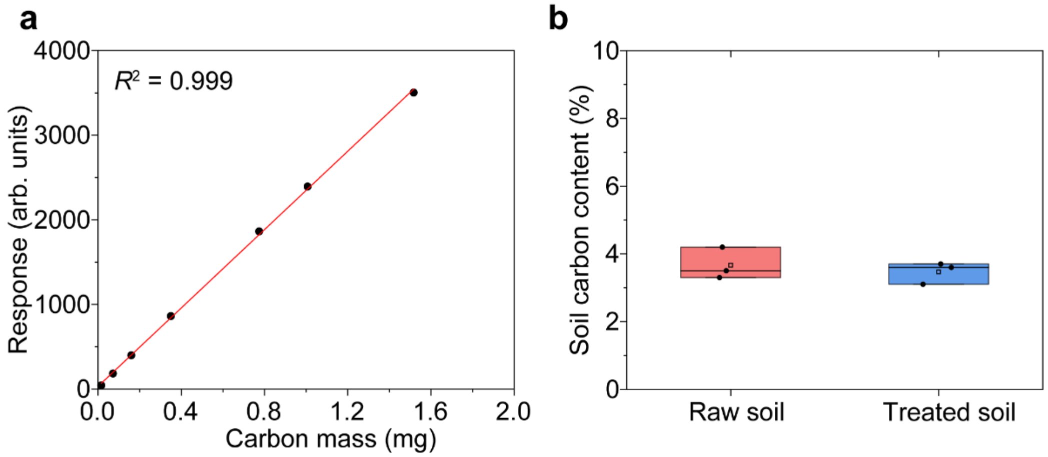

Supplementary Fig. 12. Soil carbon content measurement. (**a**) Calibration curve for the soil carbon content measurement. (**b**) Soil carbon content in the raw soil and the treated soil after removal of the carbon additives by sieving.

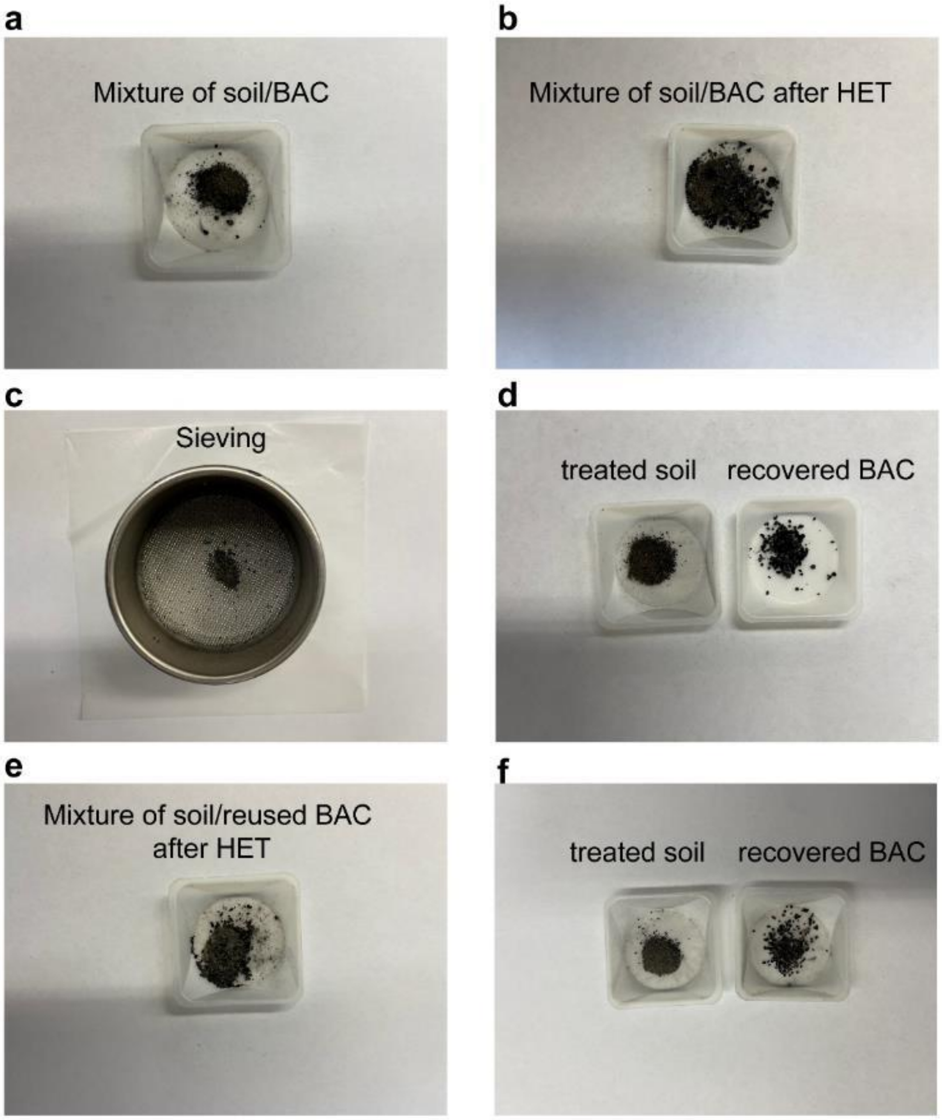

Supplementary Fig. 13. Separation and reuse of the carbon additive, bituminous activated charcoal (BAC). (**a**) Picture of the mixture of soil and BAC. (**b**) Picture of the mixture of soil and BAC after high-temperature electrothermal process (HET). (**c**) The separation of soil and BAC by sieving. (**d**) Picture of the separated treated soil and recovered BAC. (**e**) Picture of the mixture of soil and reused BAC after HET. (**f**) Picture of the separated treated soil and recovered BAC.

A mixture of soil (~200 mg) and BAC (~100 mg) were used for the HET process. In a typical experiment, the recovered mass of BAC was *m*(recovered BAC) = 95.5 mg, resulting in a

BAC recovery yield of ~95.5%. The recovered BAC could be reused for further HET treatment. To demonstrate this, we used the recovered BAC (95.5 mg) with some new BAC (4.5 mg) as the conductive additives to purify another batch of soil (200 mg). After the HET process and subsequent separation by sieving, we recovered the BAC with a mass of *m*(recovered BAC) = 93.2 mg, resulting in a BAC recovery yield of ~93.2%.

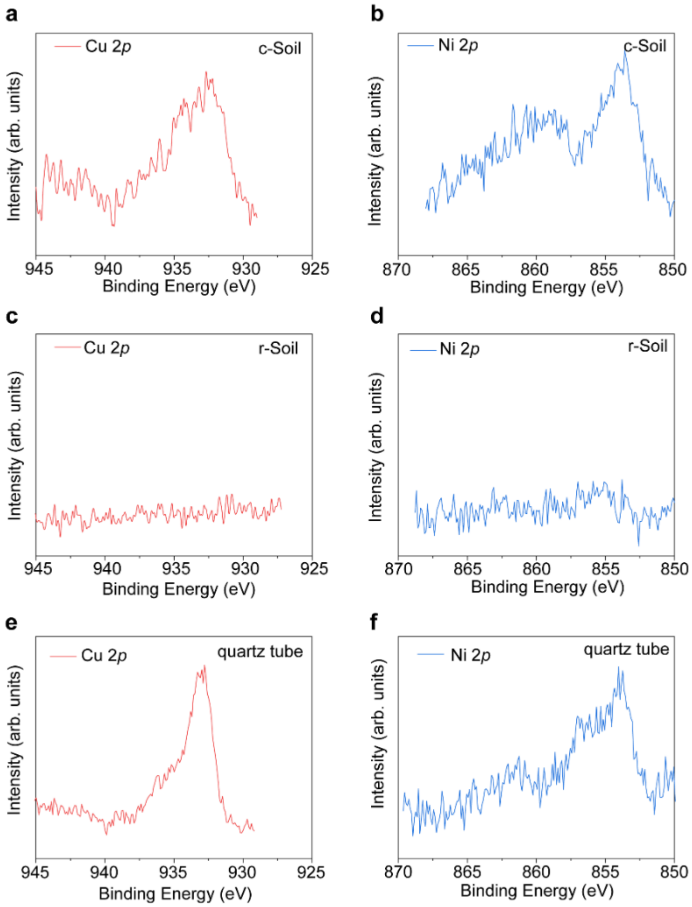

Supplementary Fig. 14. XPS characterization of heavy metals. (**a, b**) XPS fine spectrum of Cu (**a**) and Ni (**b**) for contaminated soil (c-Soil). (**c, d**) XPS fine spectrum of Cu (**c**) and Ni (**d**) for remediated soil (r-Soil). (**e, f**) XPS fine spectrum of Cu (**e**) and Ni (**f**) of quartz tube sidewall after HET.

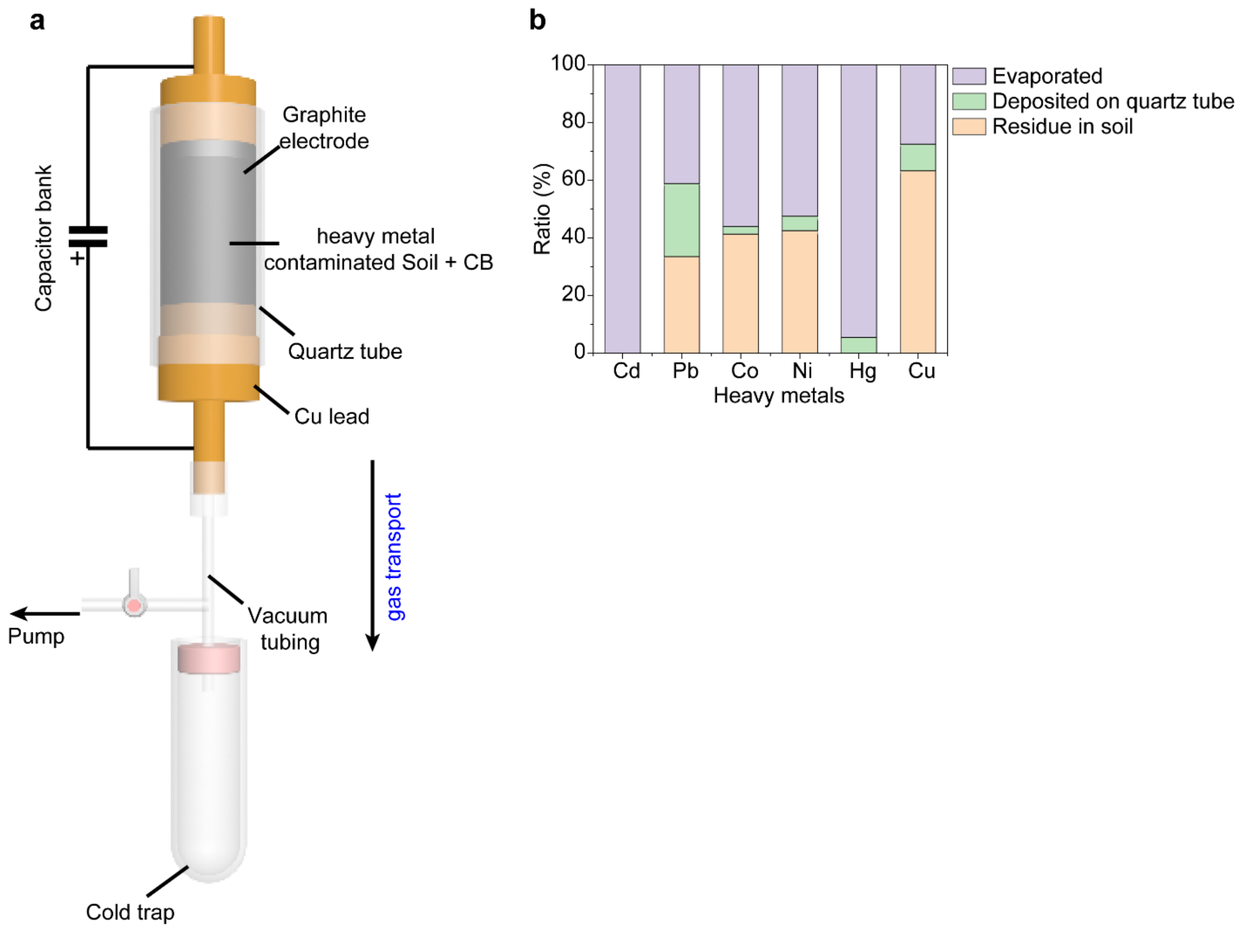

Supplementary Fig. 15. Heavy metals mass balance measurement. (**a**) Schematic of the vacuum apparatus for collecting the volatiles during HET. (**b**) Mass balance of heavy metals.

We conducted heavy metals mass balance measurements using a HET voltage of 80 V for a single electric pulse. While this condition was not optimized, it allows us to quantify the distribution of heavy metals during the HET process. We found that heavy metals could be deposited on the quartz tube, evaporated, or remain in the residual soil. We calculated the amounts of heavy metals in the residual soil and on the quartz tube, and considered the remaining portion as the evaporated fraction.

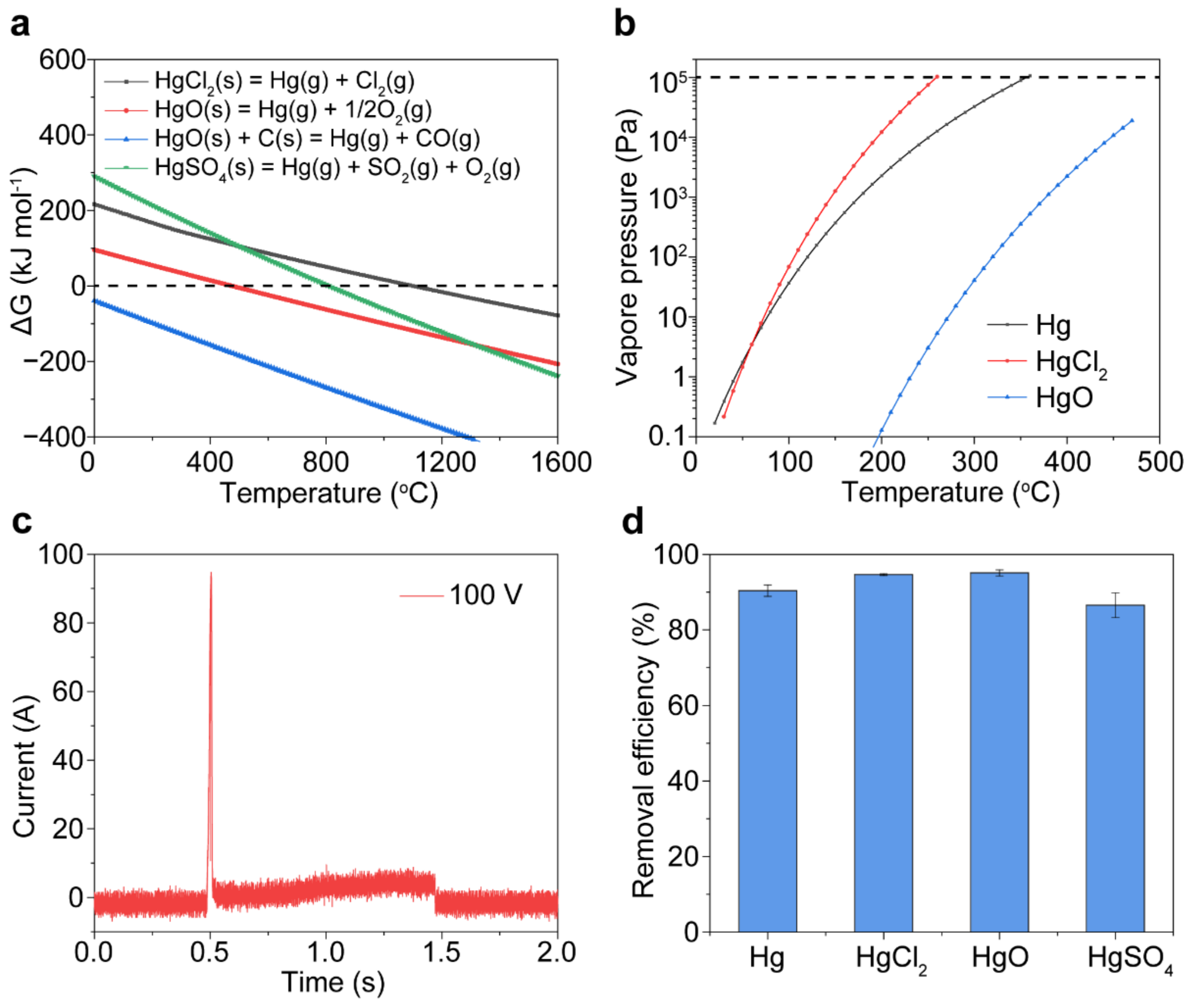

Supplementary Fig. 16. Remediation of soil contaminated with various Hg species. (**a**) Gibbs free energy changes of the reactions. The dash line denotes ΔG = 0 kJ mol^{-1}. (**b**) Vapor pressure-temperature relationship for various Hg species. The dash line denotes vapor pressure of 1 atm. (**c**) Current input for the remediation of Hg-contaminated soil. (**d**) Removal efficiencies of different Hg species with a single HET pulse.

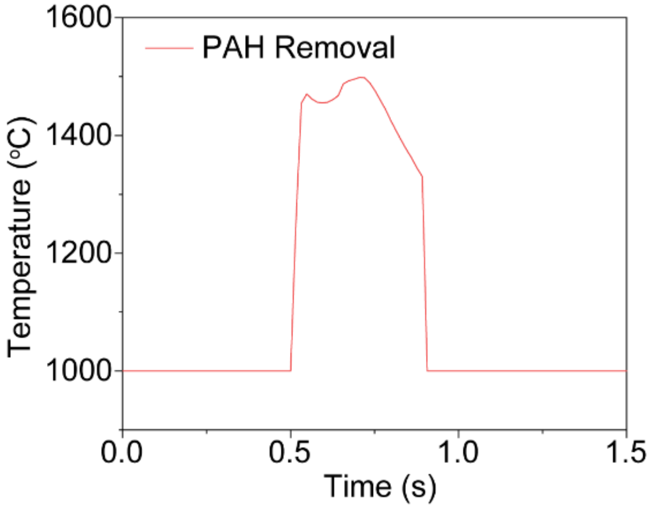

Supplementary Fig. 17. Temperature measurement for polycyclic aromatic hydrocarbon (PAH) remediation. Temperature profile of HET at voltages of 100 V.

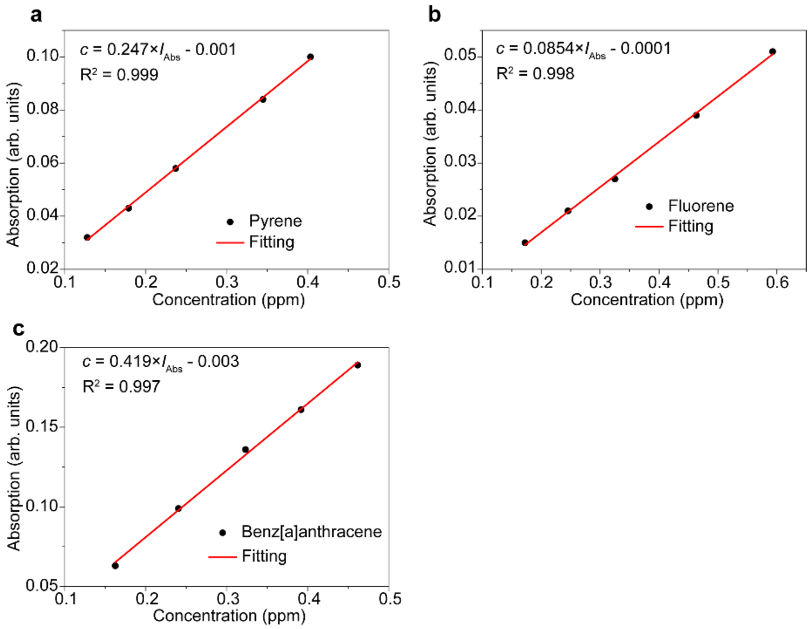

Supplementary Fig. 18. Calibration curves for PAH by UV-Vis spectrophotometry. (**a**) Calibration curve of pyrene. (**b**) Calibration curve of fluorene. (**c**) Calibration curve of benz[a]anthracene. The linearity of the fitting is good ($R^2 > 0.99$) for all three cases, demonstrating the validity of the method for concentration quantification.

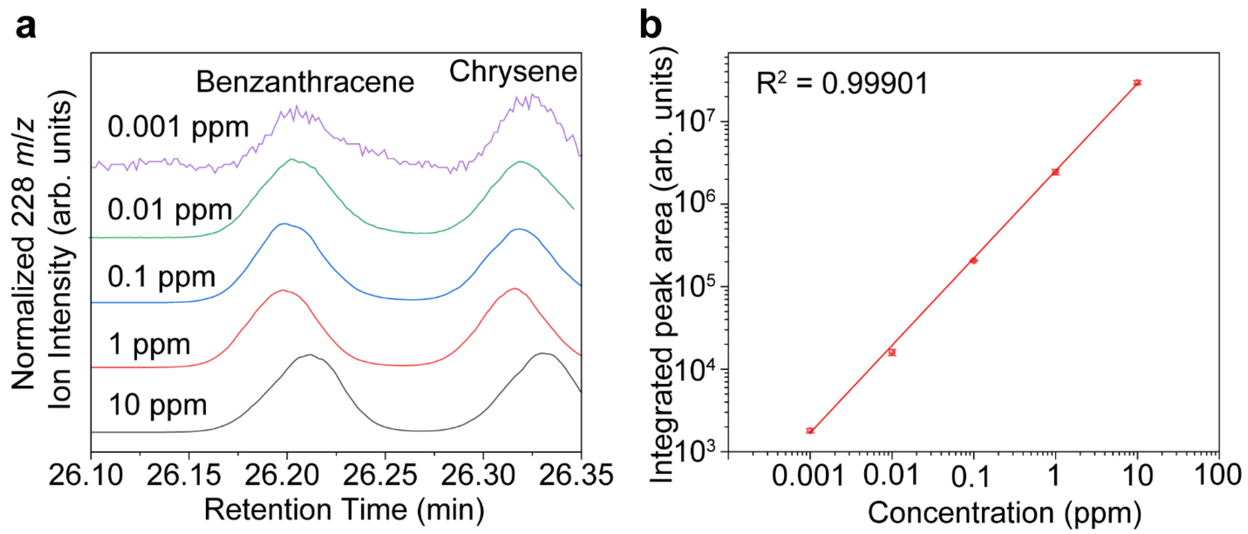

Supplementary Fig. 19. Calibration curves for benz[a]anthracene by GC-MS. (**a**) Chromatogram of calibration curves. The peak of chrysene is also shown, which is distinguished by deuteration. (**b**) Calibration curve of benz[a]anthracene, showing >0.001 ppm limit of detection. The linearity of the fitting is good (R^2 >0.999), demonstrating the validity of the method for concentration quantification. The error bars denote SD where N = 3.

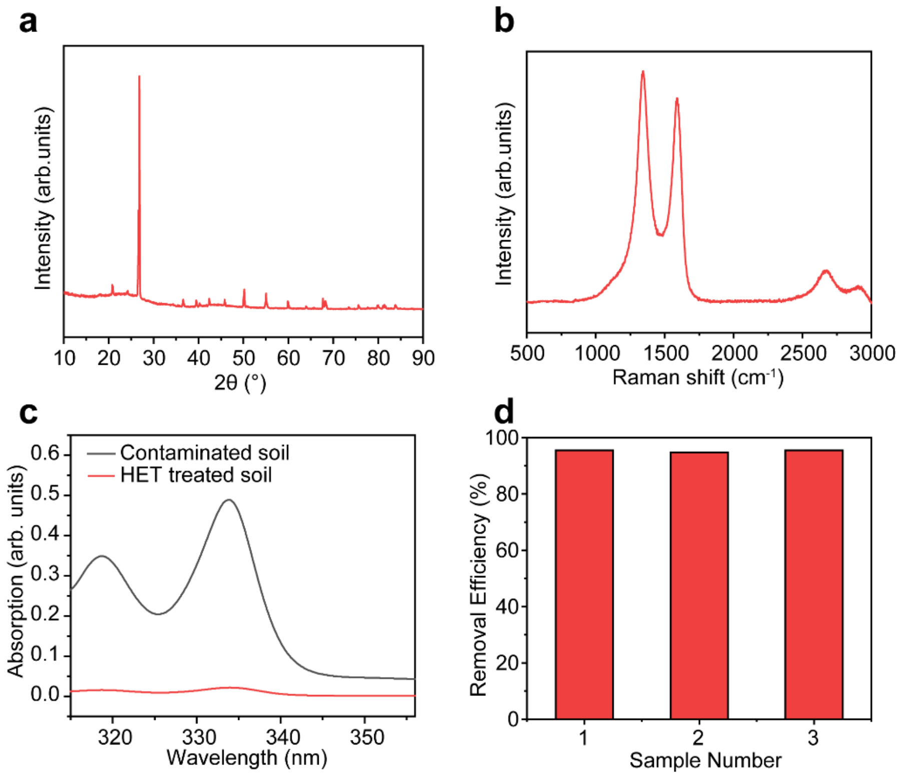

Supplementary Fig. 20. Remediation of pyrene-contaminated soil using biochar as conductive additive. (**a**) XRD pattern of biochar. (**b**) Raman spectrum of biochar. (**c**) UV-Vis absorption spectra of extracts from pyrene-contaminated soil and HET treated soil. (**d**) Removal efficiencies of pyrene.

Biochar with sufficient conductivity could also be used as the conductive additive. Biochar is primarily composed of carbon with some inorganic constituents (Supplementary Figs. 20a-b). The contaminated soil was mixed with biochar at a mass ratio of 2:1. The HET treatment conditions for this mixture remained the same as those used for carbon black additives (Supplementary Table 2). Following the HET treatment, the pyrene content was significantly reduced (Supplementary Fig. 20c). The removal efficiency of pyrene achieved by a single HET pulse reached ~95% (Supplementary Fig. 20d), which is comparable to the performance observed with other carbon additives (Fig. 3).

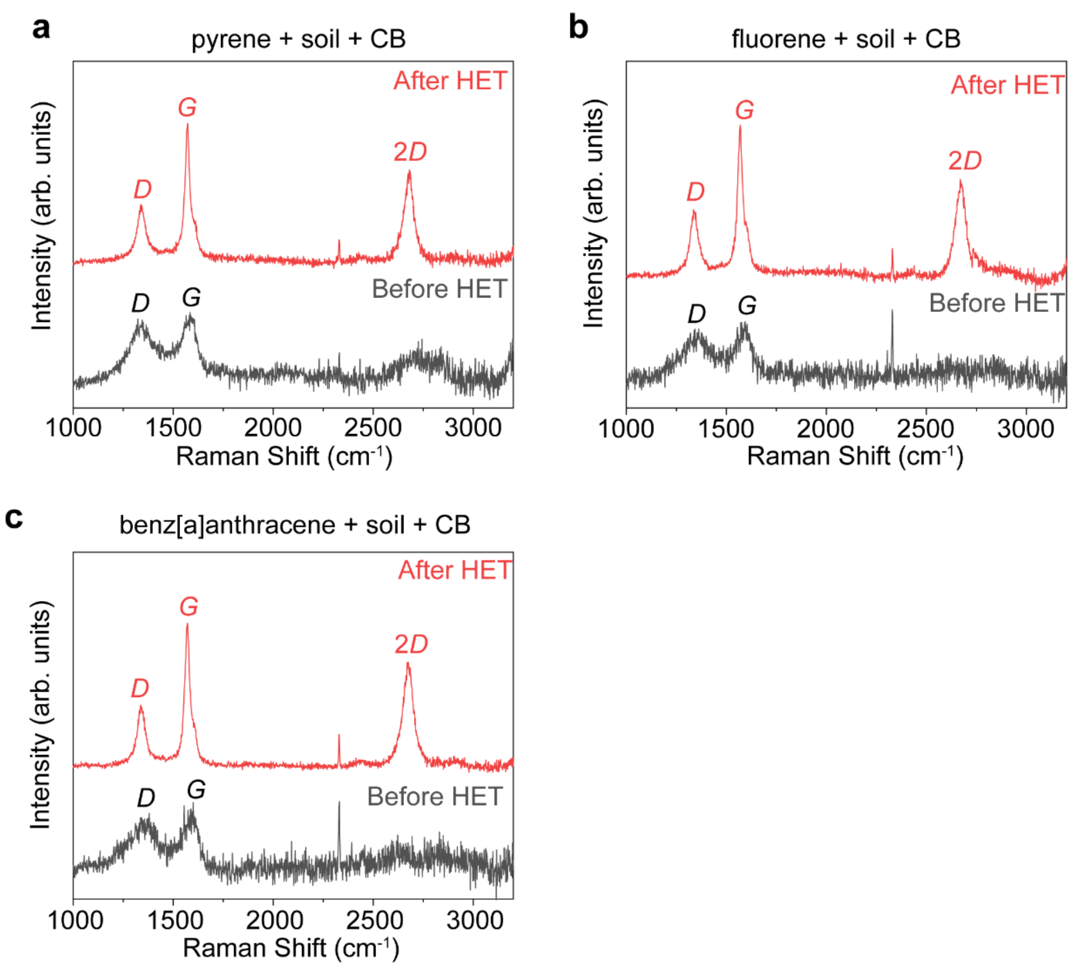

Supplementary Fig. 21. Raman spectra of PAH-contaminated soil mixed with carbon additives before and after HET treatment. (**a**) Raman spectra of the mixture of pyrene, soil, and carbon black (CB) before and after high-temperature electrothermal process (HET). (**b**) Raman spectra of the mixture of fluorene, soil, and CB before and after HET. (**c**) Raman spectra of the mixture of benz[a]anthracene, soil, and CB before and after HET.

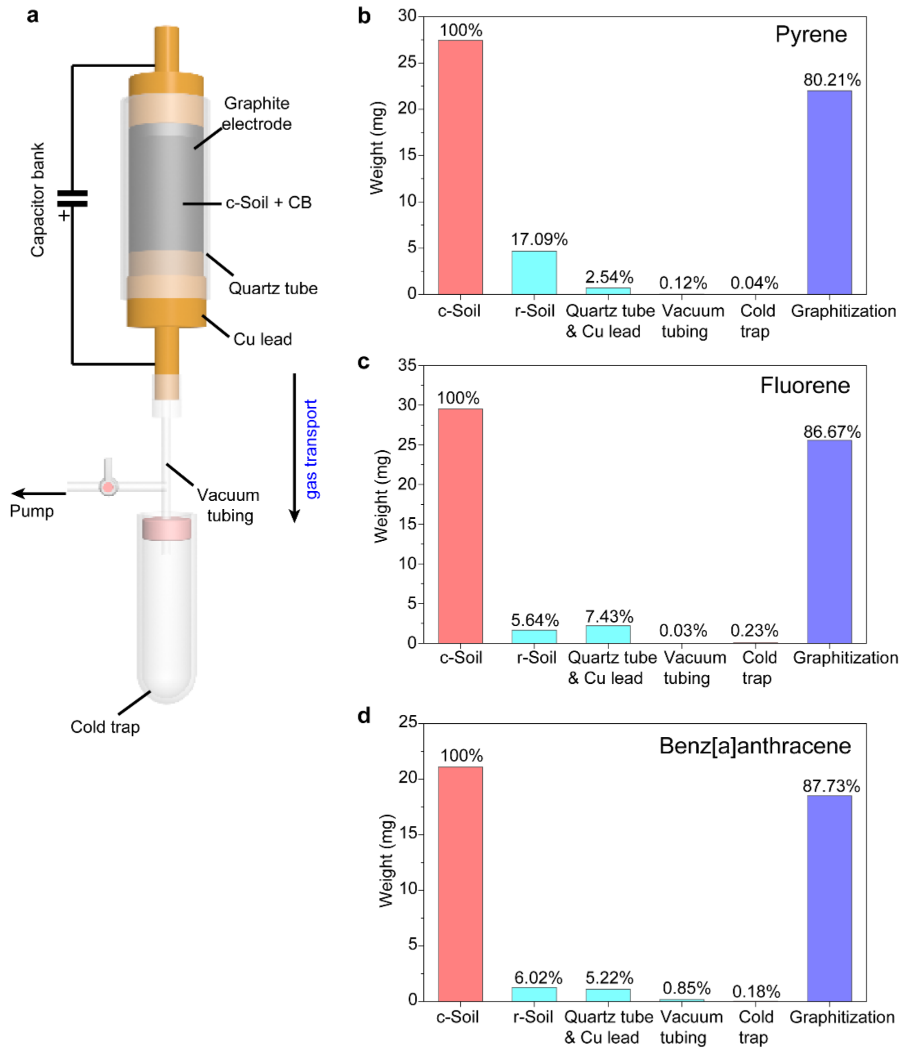

Supplementary Fig. 22. PAH mass balance measurement. (**a**) Schematic of the apparatus to capture evaporated PAH. (**b**) Pyrene weight in contaminated soil (c-Soil), remediated soil (r-Soil), quartz tube and Cu lead, vacuum tubing, and cold trap after one HET pulse. The unaccounted amount is considered as graphitized part. The percentages of each are labeled. (**c**) Fluorene weight in c-Soil, r-Soil, quartz tube and Cu lead, vacuum tubing, and cold trap after one HET pulse. The unaccounted amount is considered as graphitized part. The percentages of each are labeled. (**d**) Benz[a]anthracene weight in c-Soil, r-Soil, quartz tube and Cu lead, vacuum tubing, and cold trap after one HET pulse. The unaccounted amount is considered as graphitized part. The percentages of each are labeled.

To determine whether the PAH are removed from the soil by graphitization or evaporation, we constructed an apparatus to collect the evaporative PAH during the HET process. The setup, shown in Supplementary Fig. 22a, consisted of a cold trap connected to the HET chamber via a vacuum tubing with a porous Cu lead to permit gas diffusion. The cold trap was first pumped to vacuum. Then, the HET was conducted, with the unreacted PAH remaining in the r-Soil, or evaporated and condensed on the quartz tube, the Cu lead, the vacuum tubing, or in the cold trap. The vacuum was released, and the PAH weights in the r-Soil, quartz tube and Cu lead, vacuum tubing, and cold trap were measured by the same solvent extraction process. The unaccounted PAH weight was considered as the graphitized fraction. As shown in Supplementary Figs. 22b-d, most of the PAH were graphitized, demonstrating that the organic contaminants in the contaminated soil were predominantly removed through graphitization rather than evaporation.

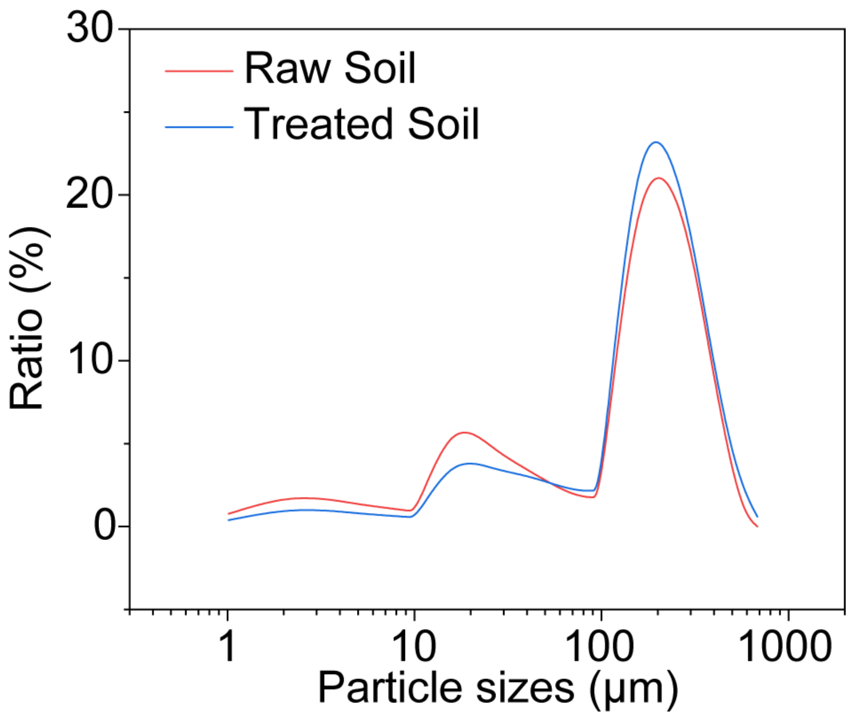

Supplementary Fig. 23. Particle size distribution measurement. The particle size distribution of raw soil and HET treated soil measured using laser particle size analyzer.

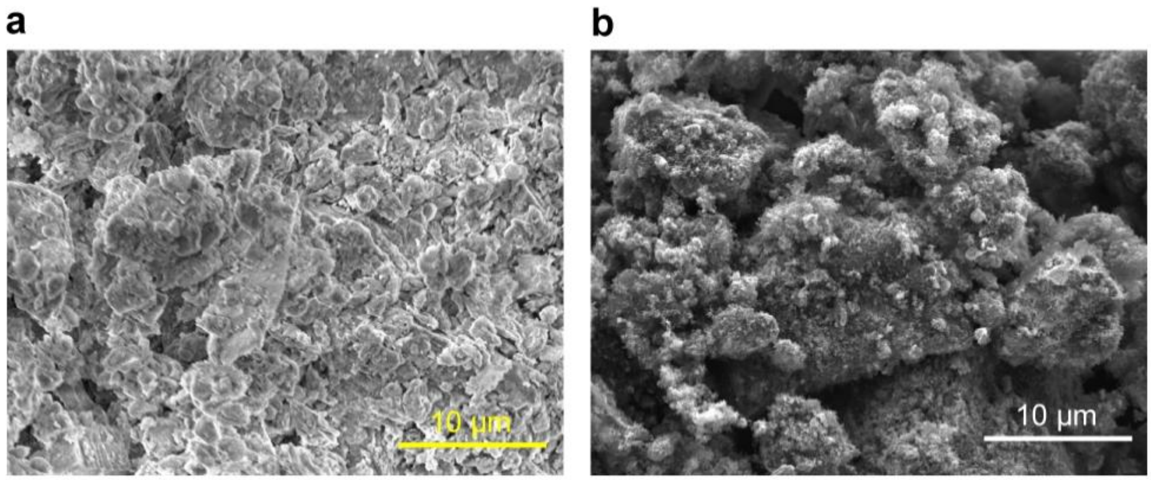

Supplementary Fig. 24. Morphology characterization. (**a**) SEM image of raw soil. (**b**) SEM image of treated soil.

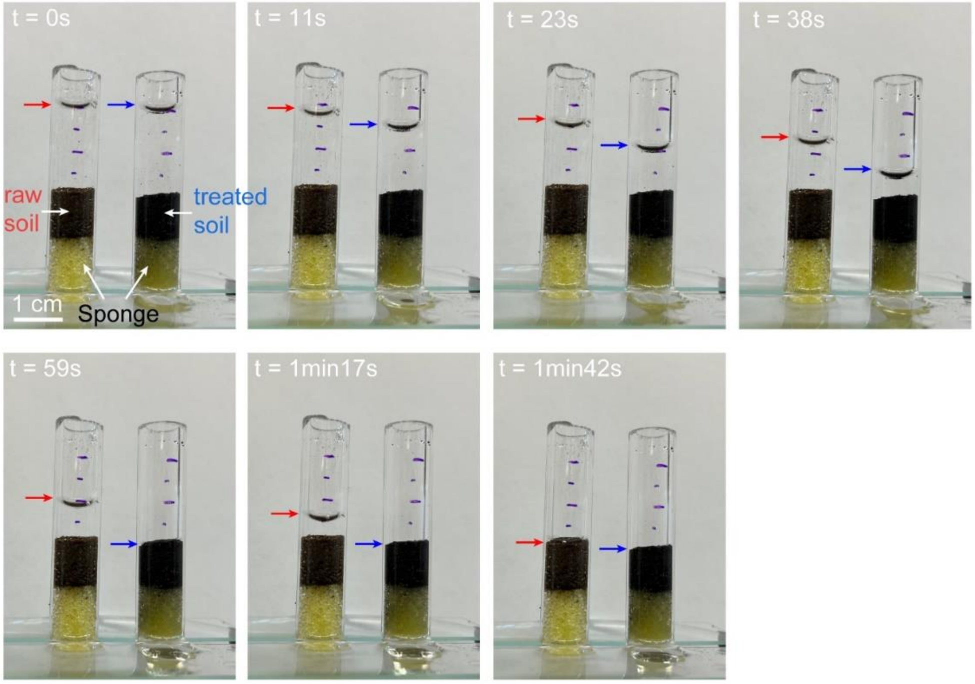

Supplementary Fig. 25. Water infiltration test of the raw soil and the soil after HET. Pictures of water levels with time. The red arrow and blue arrow show the liquid levels of raw soil and the soil after HET, respectively.

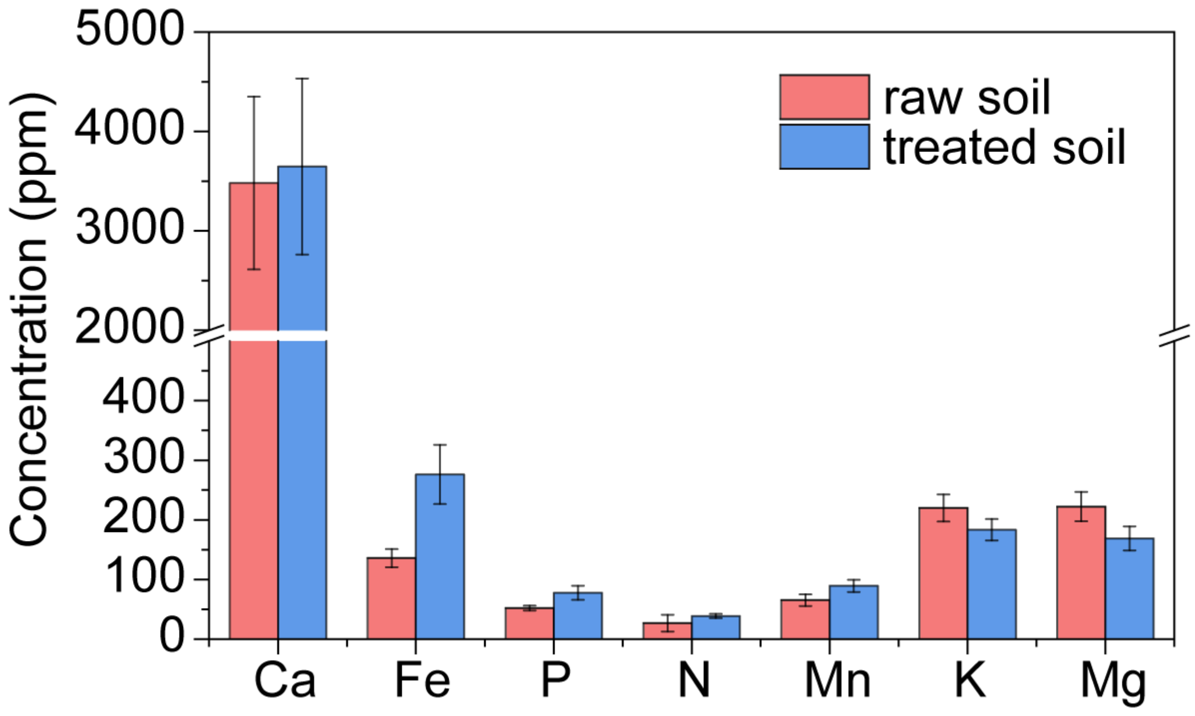

Supplementary Fig. 26. Soil nutrients concentration measurement. The exchangeable contents of Ca, Fe, P, N, Mn, K, and Mg in the raw soil and HET treated soil. The error bars denote SD where N = 3.

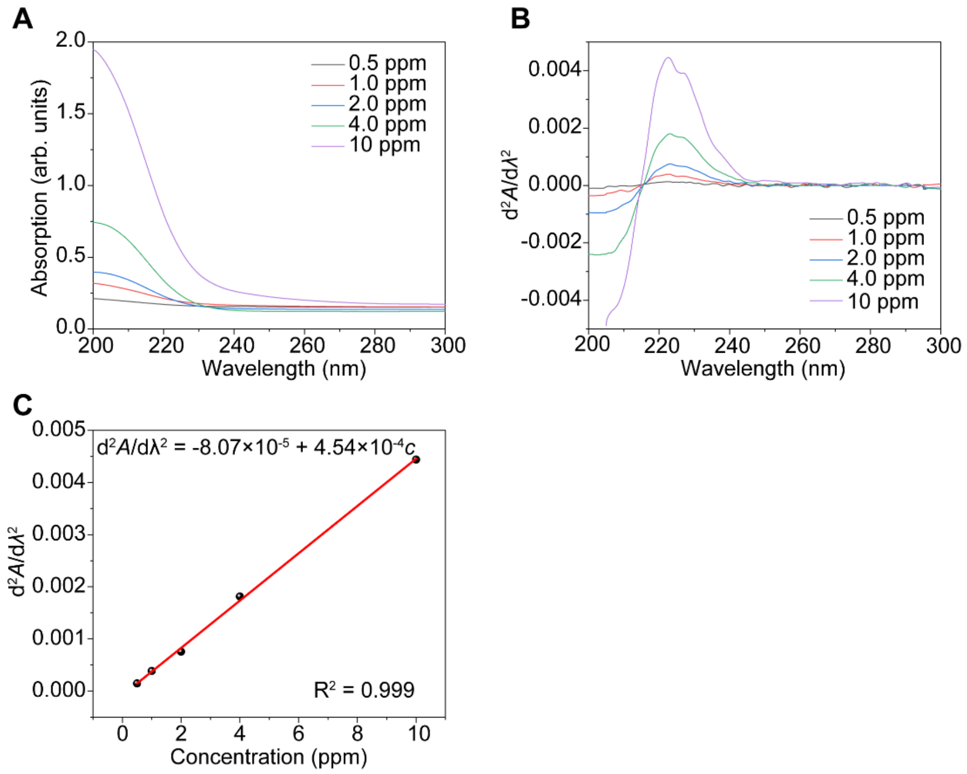

Supplementary Fig. 27. Measurement of nitrate-nitrogen in soil by ultraviolet secondary-derivative spectrophotometry. (**a**) UV spectra of nitrate solution ($NaNO_3$) with varied concentrations. (**b**) Secondary-derivative spectra of nitrate solution with varied concentrations. (**c**) Standard curve for the nitrate by using the secondary derivative absorption at 223 nm.

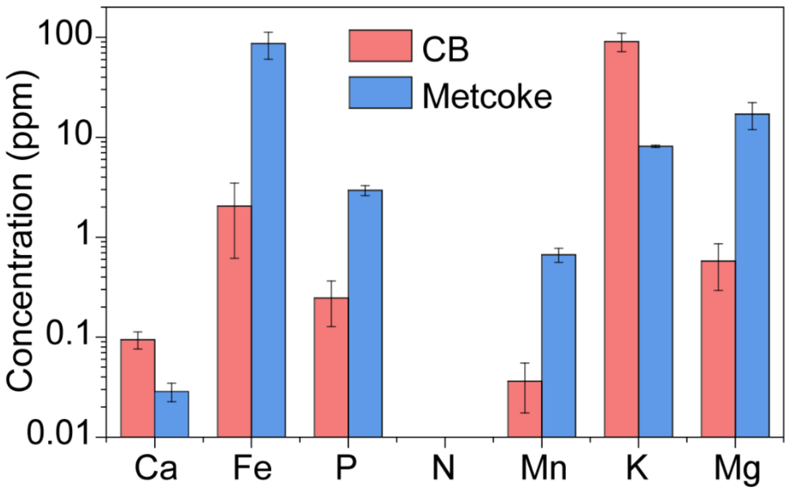

Supplementary Fig. 28. Exchangeable nutrients measurement in carbon additives, including CB and Metcoke. Note that N content is lower than 0.1 ppm (the detection limit of UV-Vis spectrometry). The error bars denote SD where N = 3.

To measure the content of exchangeable nutrients in the conductive additives, such as carbon black (CB) and Metcoke, we followed the same measurement process as with the soil samples. The concentrations of various nutrients in CB are (values in ppm): Ca(0.09), Fe(1.3), P(0.14), N(<0.1), Mn(0.02), K(69), and Mg(0.37). In Metcoke, the concentrations are (values in ppm): Ca(0.02), Fe(85), P(0.14), N(<0.1), Mn(0.02), K (69), and Mg(0.37). Let us consider Metcoke as an example. Taking into account the ~3% carbon residue in the treated soil, the nutrients introduced by the carbon additives are (values in ppm): Ca(0.0006), Fe(2.55), P(0.0042), N(<0.003), Mn(0.00006), K(2.1), and Mg(0.011). For comparison, the nutrient concentrations in the raw soil are (values in ppm): Ca(4474), Fe(152), P(56.7), N(10.6), Mn(76.6), K(245), and Mg(250). Therefore, the measurement errors introduced by the residual carbon are: Ca(1.3×10-5%), Fe(1.7%), P(7.4×10-3%), N(<0.028%), Mn(7.8×10-5%), K(0.86%), and Mg(4.4×10-3%). In conclusion, the carbon additives have no significant effect on the exchangeable nutrient concentrations.

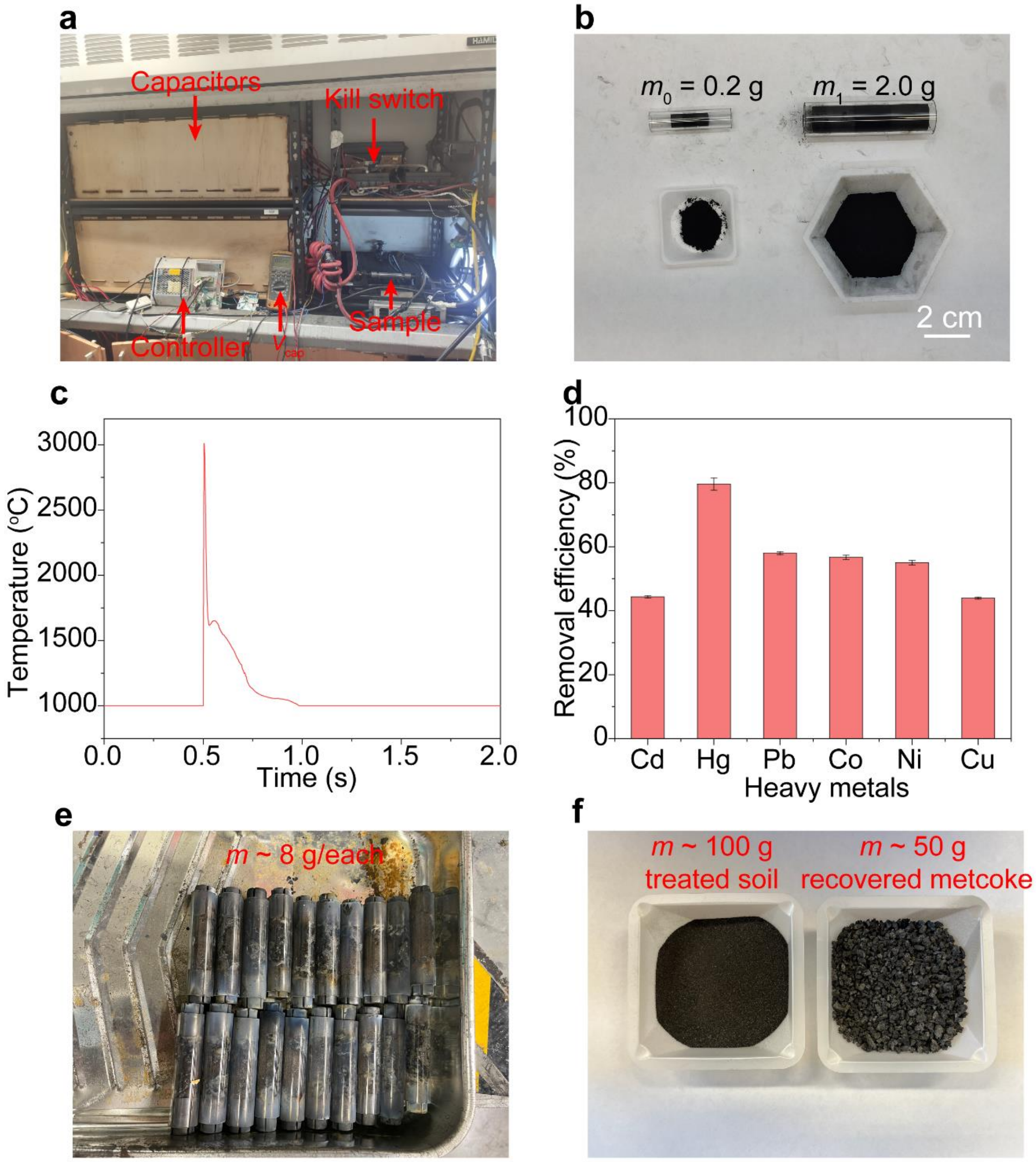

Supplementary Fig. 29. Scaling up of the HET process for soil remediation. (**a**) Picture of the large-scale HET equipment with C_1 = 0.624 F filling a 1.5 m-long hood. (**b**) Picture of the samples. (**c**) Real-time temperature curve of the large-scale sample. (**d**) Heavy metal removal efficiencies. The error bars denote SD where N = 3. (**e**) 20 treated samples with each sample mass of ~8 g by using Metcoke as the conductive additive. (**f**) Treated soil with mass of ~100 g and the recovered Metcoke of ~ 50 g.

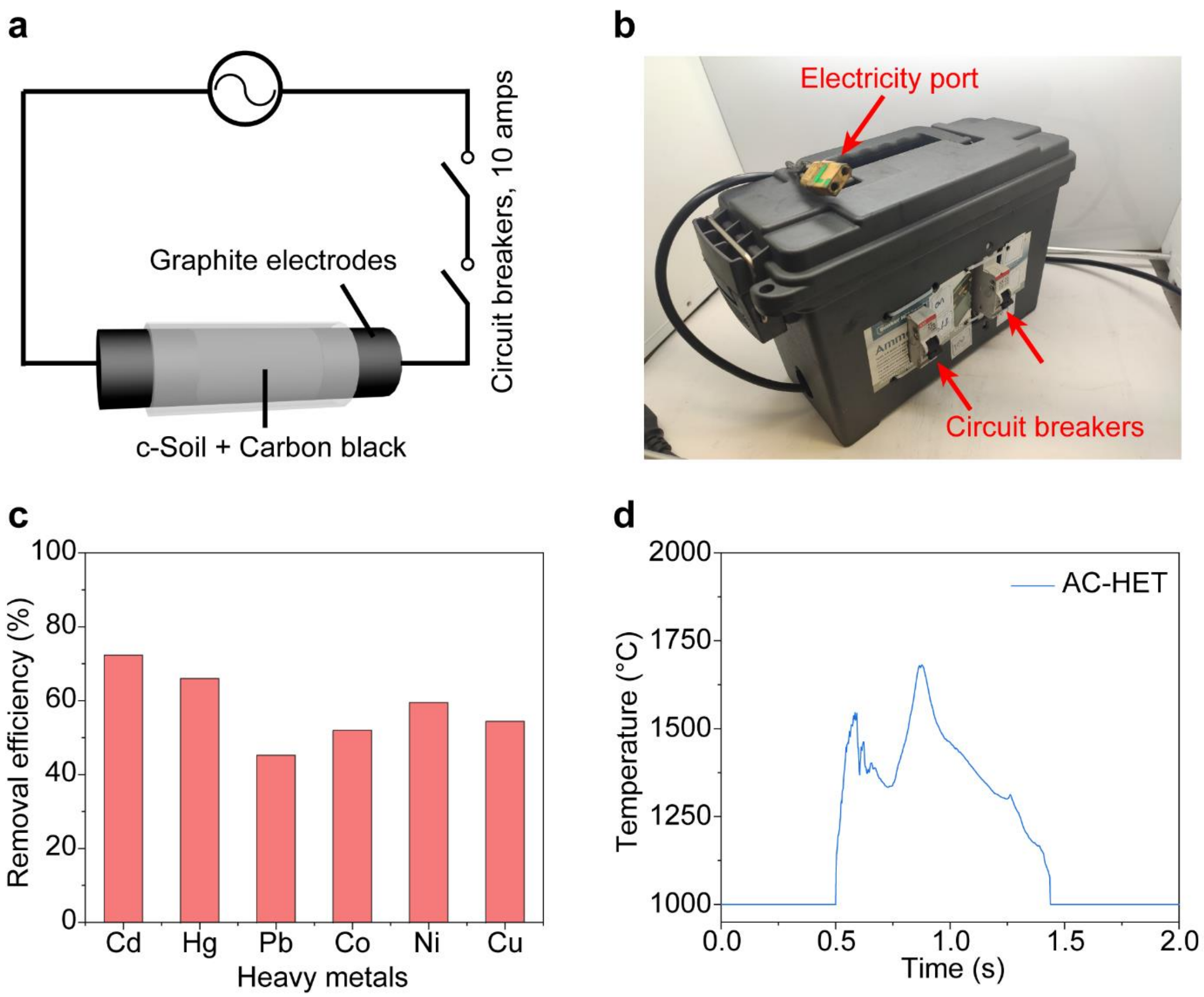

Supplementary Fig. 30. The heavy metal removal using AC-HET system. (**a**) The schematic of the AC-HET system. Two circuit breakers (maximum current of 10 A) were used. c-Soil, contaminated soil. (**b**) The picture of the AC-HET system. (**c**) Removal efficiencies of heavy metals by AC-HET. (**d**) Temperature measurement of the alternating current high-temperature electrothermal process (AC-HET).

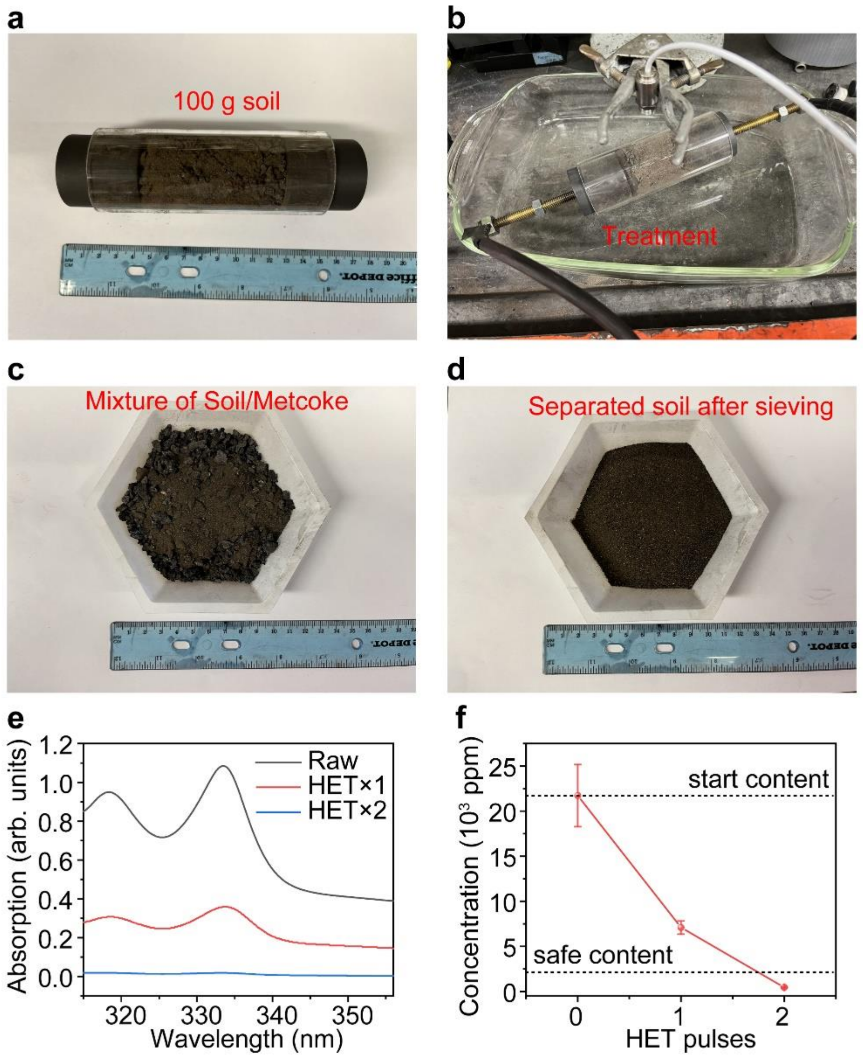

Supplementary Fig. 31. Scaling up sample mass to 100 g per batch using AC system. (**a**) Picture of the 100 g soil sample mixed with metallurgical coke (Metcoke) as conductive additives. (**b**) Picture of the sample for HET treatment. (**c**) Picture of the mixture of soil/Metcoke after the high-temperature electrothermal process (HET) treatment. (**d**) Separated soil after sieving process. (**e**) UV-Vis absorption spectra of extracts from pyrene-contaminated soil before and after repetitive HET treatment. (**f**) The content of pyrene in soil varied with repetitive HET treatment. The safe contents denote the preliminary remediation goals[29]. The error bars denote SD where N = 3.

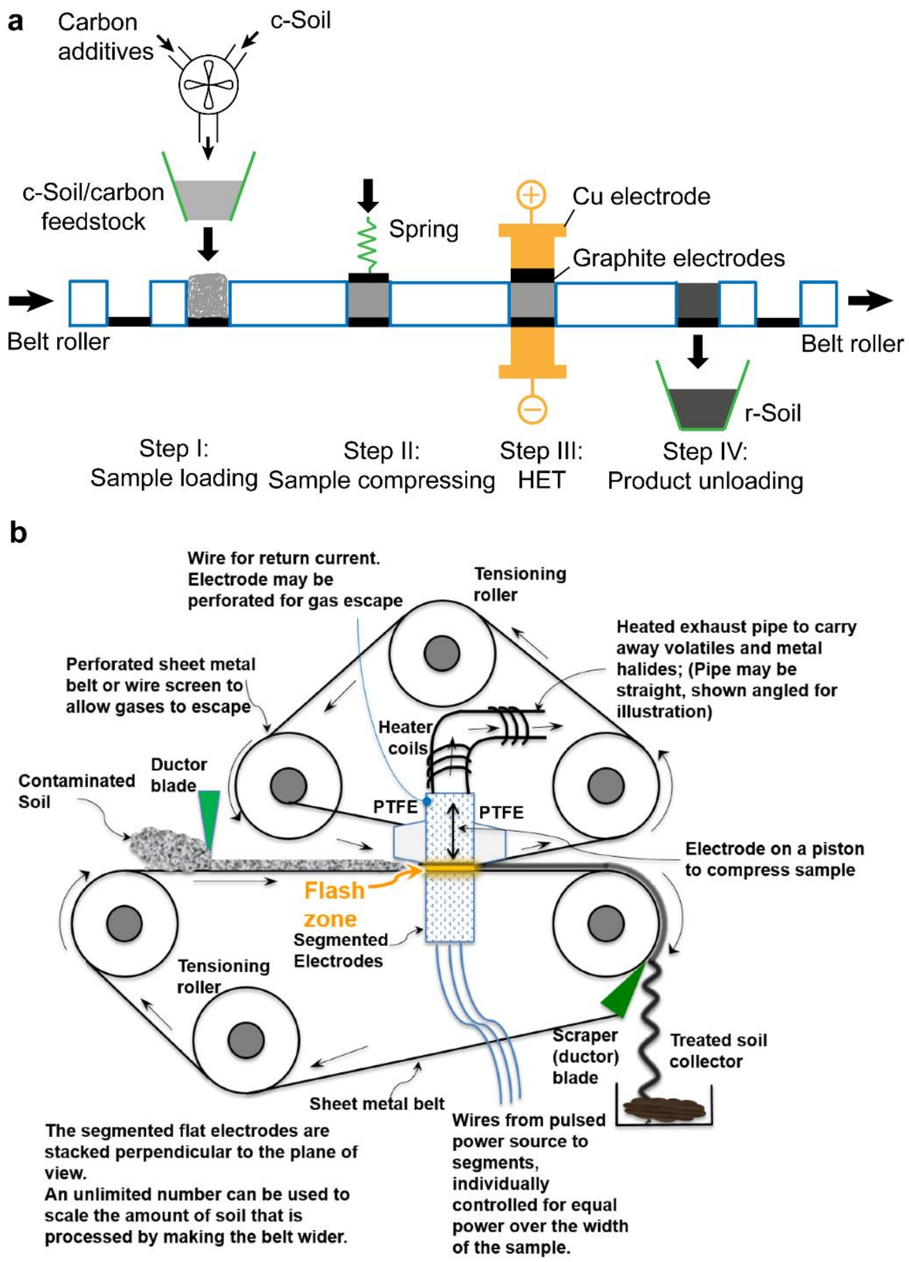

Supplementary Fig. 32. Conceptual designs of the continuous HET processing. (**a**) Scheme of the design for continuous high-temperature electrothermal process (HET) using a straight belt roller. c-Soil, contaminated soil; r-Soil: remediated soil. (**b**) Scheme of the design for continuous HET using a circulating belt roller. The soil can be excavated from the contaminated sites using excavators. The soil and carbon additives can be mixed using a commercial mixer. PTFE, polytetrafluoroethylene.

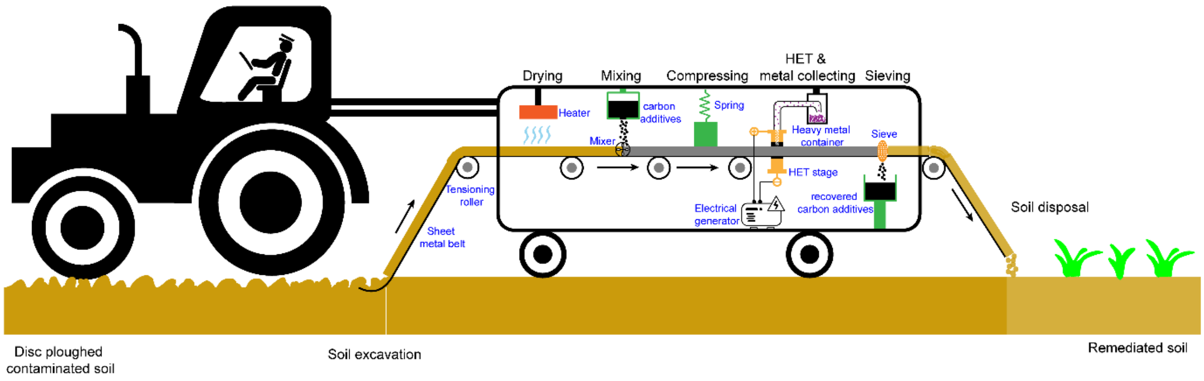

Supplementary Fig. 33. Conceptual design of a tractor attached HET unit for on-site soil remediation.

The process involves the following steps: (1) Disc ploughing the soil to soften it. The depth of soil is determined by the disc ploughing machine with typical penetration depth ranging from several to tens of centimeters. (2) Excavating and converting the contaminated soil using a sheet metal belt and tensioning roller system. (3) Drying the soil to reduce moisture content, if necessary. (4) Adding carbon additives and mixing them with the dried soil using a mixer. (5) Compressing the mixture to an appropriate resistance. (6) Joule heating the mixture using electricity provided by the generator and collecting the heavy metal volatiles in a trap. (7) Separating the carbon additives from the soil by sieving. (8) Redepositing the remediated soil at nearly its original location. HET, high-temperature electrothermal process.

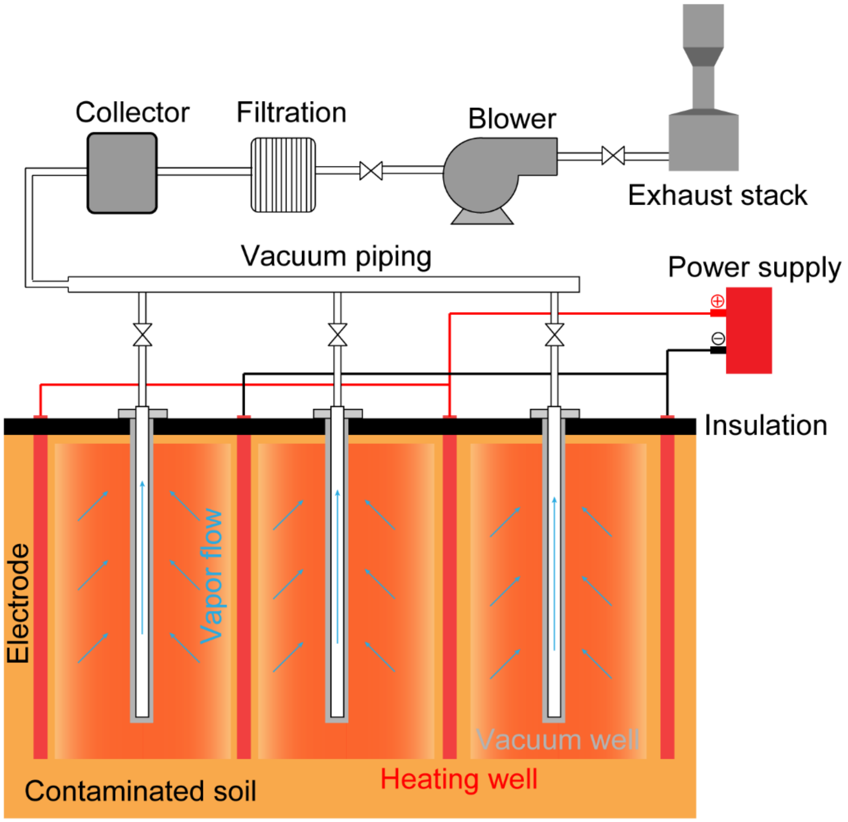

Supplementary Fig. 34. A conceptual design of a field facility for on-site soil remediation. The facility includes a vacuum well, vacuum piping, collector, filtration, blower, and exhaust stack. In this design, the contaminated soil is considered as a dry, porous material. Soil is firstly mixed with carbon conductive additives. The electrodes are installed in the heating well, with the depth determined by the level of contamination. A vacuum collection system is designed to capture the volatile matter during the HET process. This design is modified from a known method[5], but in our case the electrodes provide a rapid voltage pulse for electric heating rather than long-duration heat injection. The depth of soil is determined by the electrodes inserted into the soil and the vacuum well. This facility is adapted from traditional in-situ thermal desorption technologies, which allow for the remediation of soil depths ranging from 4.5 to 6.5 m, as mentioned in the reference[30].

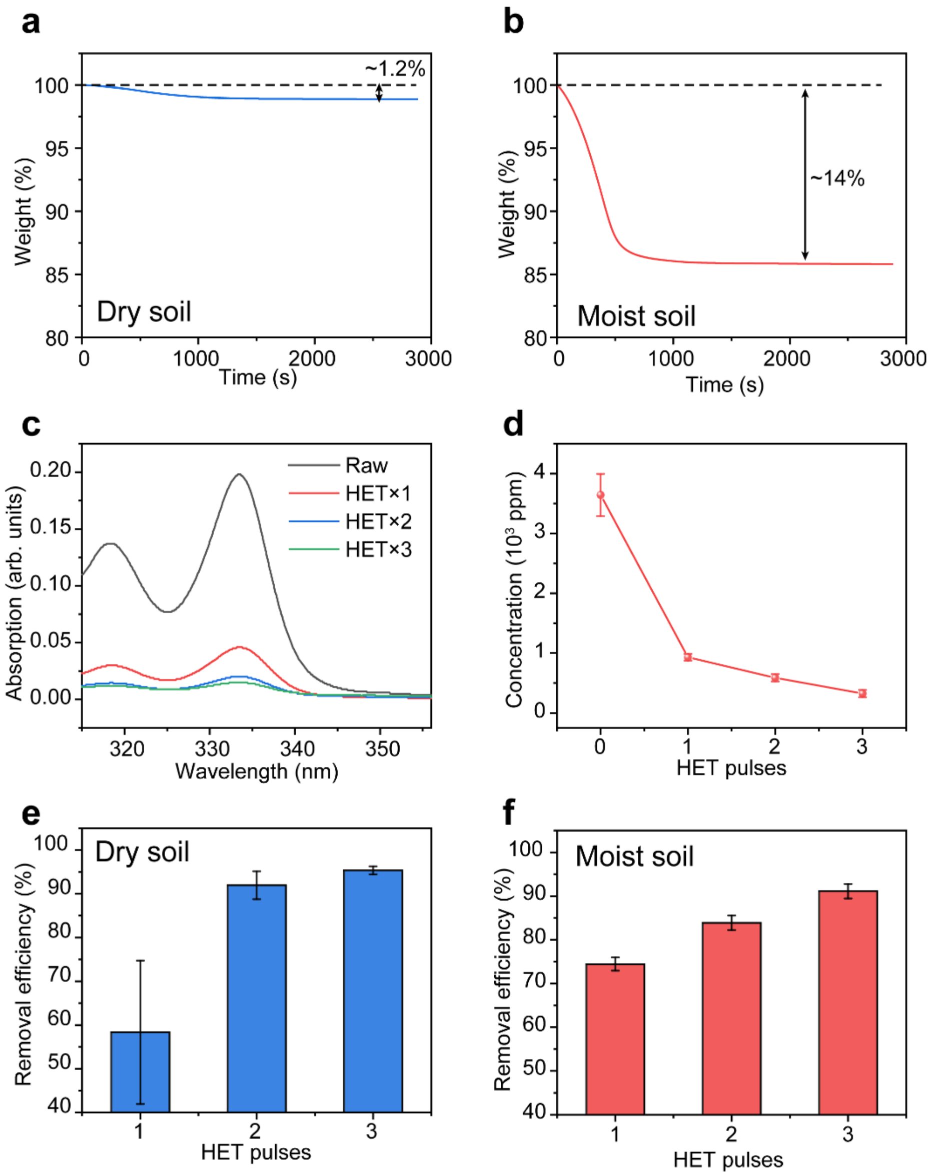

Supplementary Fig. 35. Remediation of moist pyrene-contaminated soil. (**a**) TGA curve of dry soil. (**b**) TGA curve of moist soil. TGA was conducted in air with a heating rate of 25 °C min^{-1} and then kept at 110 °C for 30 min. (**c**) UV-Vis absorption spectra of extracts from pyrene-contaminated moist soil before and after repetitive high-temperature electrothermal (HET) pulses. (**d**) The content of pyrene in soil varied with repetitive HET pulses. (**e**) Removal efficiency of pyrene in dry soil varied with repetitive HET pulses. (**f**) Removal efficiency of pyrene in moist soil varied with repetitive HET pulses.

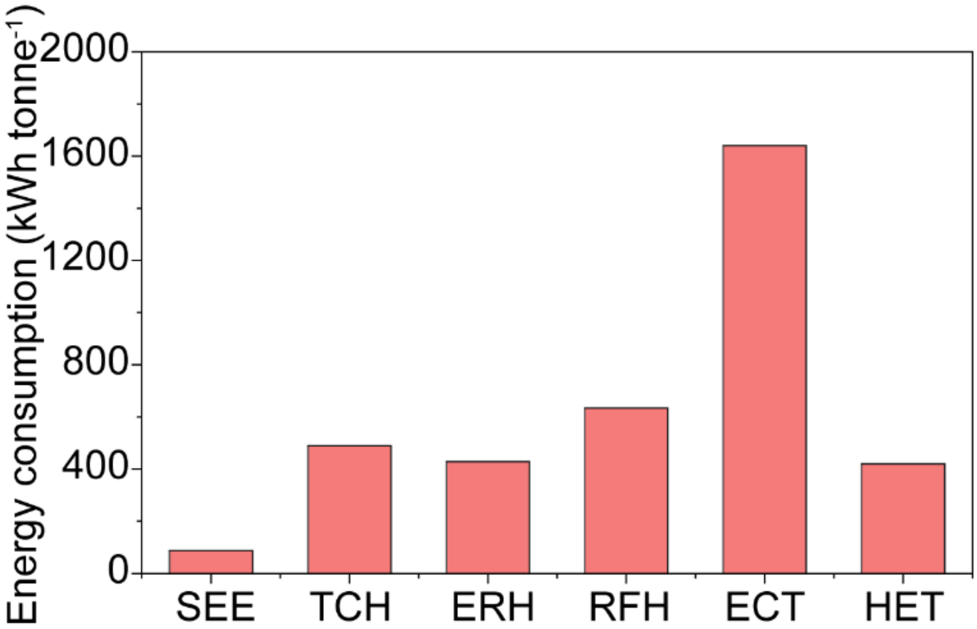

Supplementary Fig. 36. Energy consumption of some soil remediation methods. SEE, steam-enhanced extraction; TCH, thermal conduction heating; ERH, electrical resistance heating; RFH, radio frequency heating; ECT, electrochemical technology; HET, high-temperature electrothermal process.

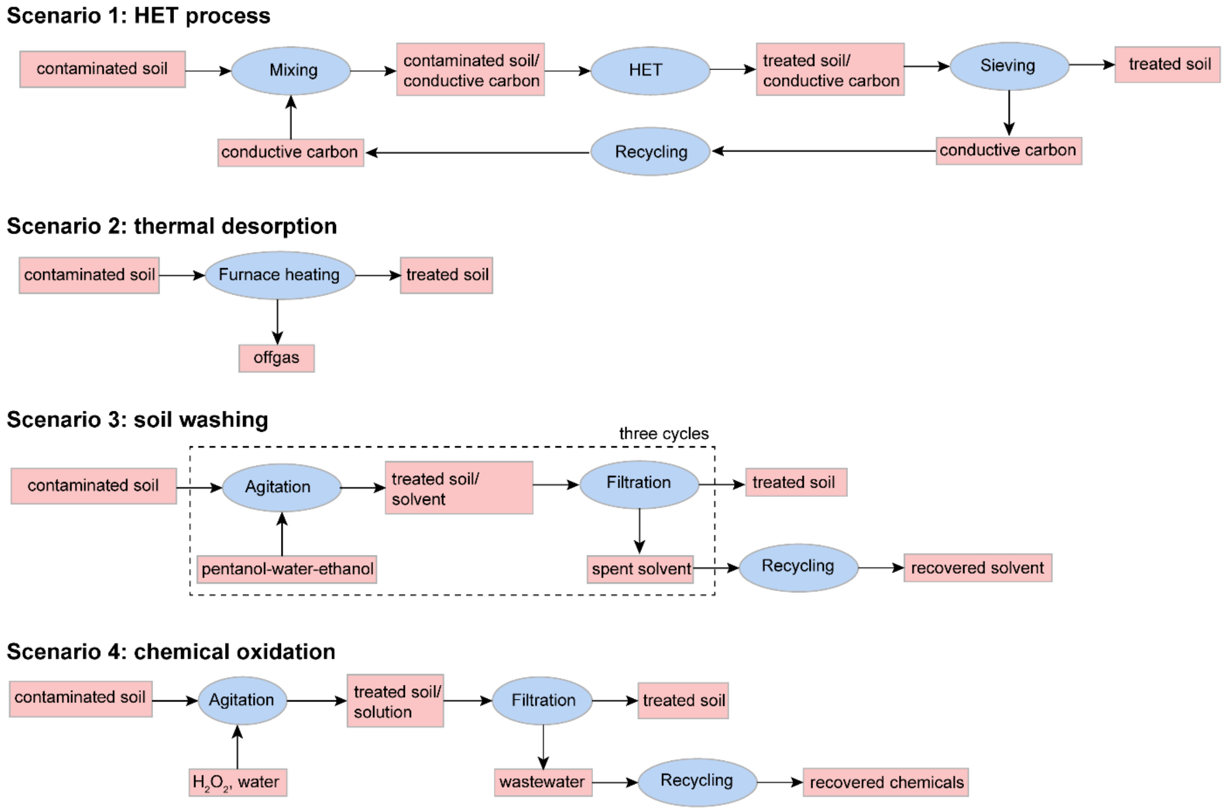

Supplementary Fig. 37. Scenarios for the life-cycle assessment and techno-economic analysis. Materials flow for high-temperature electrothermal (HET) process, thermal desorption, soil washing, and chemical oxidation.

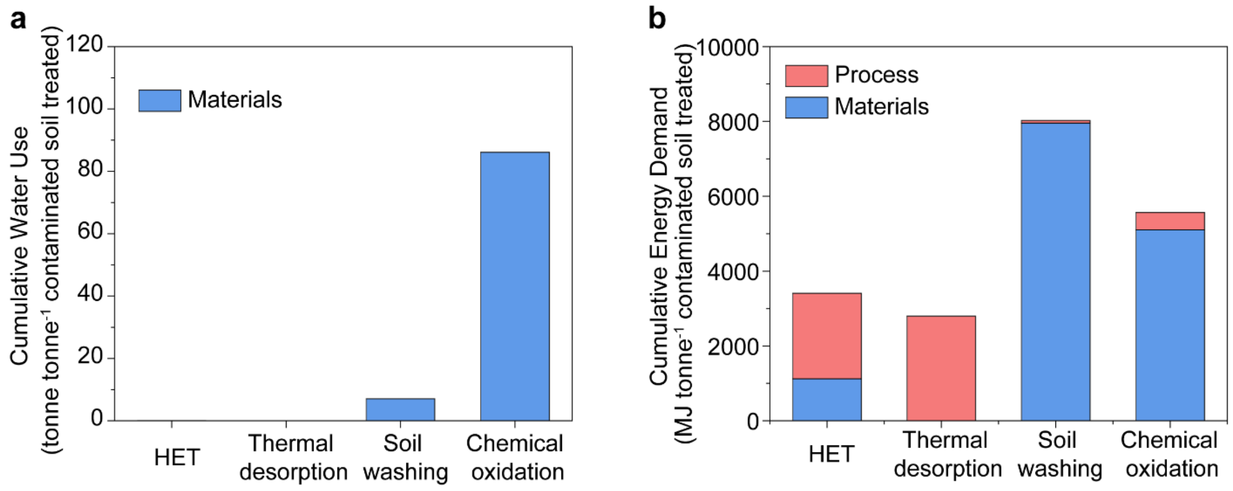

Supplementary Fig. 38. Life-cycle impact assessment. (**a**) Cumulative water use of various scenarios. (**b**) Cumulative energy demand of various scenarios. HET, high-temperature electrothermal process.

Supplementary Table 1. Physical properties of the precursors and corresponding metals/metalloid.

Precursors	Decomposition temperature (°C)	Metal	Melting point (°C)	Boiling point (°C)
$CdCl_2$	961	Cd	321	767
$HgCl_2$	138	Hg	-38.8	356.7
HgO	475			
$HgSO_4$	810			
$Pb(NO_3)_2$	200 – 470	Pb	327	1749
$CoCl_2$	>873	Co	1495	2927
$CuCl_2$	993	Cu	1085	2562
$NiCl_2$	>800	Ni	1455	2913

Supplementary Table 2. Parameters for HET.

Precursors	Mass Ratio	Mass (mg) *	Resistance (Ω)	Voltage (V)	Time (s)	Mass after HET (mg) **
c-Soil(heavy metals):CB	2:1	200	1.0	60	1	145
c-Soil(heavy metals):CB	2:1	200	1.0	80	1	105
c-Soil(heavy metals):CB	2:1	200	1.0	100	1	81
c-Soil(heavy metals):CB	2:1	200	1.0	120	1	111
c-Soil(heavy metals):Metcoke	2:1	206	2.0	100	1	122
c-Soil(heavy metals):FG	2:1	211	1.5	100	1	135
c-Soil(heavy metals):Plastic Ash	2:1	200	3.0	100	1	120
c-Soil(Hg):CB	2:1	200	1.0	100	1	141
c-Soil(HgO):CB	2:1	200	1.0	100	1	162
c-Soil($HgSO_4$):CB	2:1	200	1.0	100	1	160
c-Soil(pyrene):CB	2:1	200	0.8	100	1	129
c-Soil(pyrene):biochar	2:1	200	3.6	100	1	143
c-Soil(moisture, pyrene):CB	2:1	200	1.3	100	1	127
c-Soil(fluorene):CB	2:1	200	0.8	100	1	135
c-Soil(benz[a]anthracene):CB	2:1	200	0.8	100	1	156

Note: * The total mass of c-Soil and CB; ** The total mass of r-Soil with residual carbon.

Supplementary Table 3. Energy consumption comparison.

Method	Energy consumption (KWh tonne^{-1})	References
Steam Enhanced Extraction	87 [a]	*Ground Water Monit. Remediat.* **2013**, *33*, 38
Thermal Conduction Heating	490 [a]	*Ground Water Monit. Remediat.* **2013**, *33*, 38
Electrical Resistance Heating	429 [a]	*Ground Water Monit. Remediat.* **2013**, *33*, 38
Radio Frequency Heating	634 [a]	*Ground Water Monit. Remediat.* **2013**, *33*, 38
Electrochemical Technique	1640	*Sci. Rep.* **2018**, *8*, 3272
High-temperature Electrothermal	420	*This work*

Note: [a] The values are obtained by assuming the soil density of 1.33 g cm^{-3}.

Supplementary Table 4. Materials flow for various scenarios.

Scenarios	HET (tonne)	Thermal desorption (tonne)	Soil washing (tonne)	Chemical degradation (tonne)
Contaminated soil	1	1	1	1
Conductive carbon (Metcoke)	0.04	0	0	0
Water	0	0	1.2	85.5
H_2O_2 (30%)	0	0	0	0.322
1-pentanol	0	0	0.00976	0
ethanol	0	0	0.16096	0
Mixing (solid-solid)	1.5	0	0	0
HET process	1.5	0	0	0
Sieving	1.335	0	0	0
Furnace heating	0	1	0	0
Agitation	0	0	12.736	89.72
Filtration	0	0	12.736	89.72

Note: [a]The materials mass flow is normalized to remediation of 1 tonne of PAH-contaminated soil.

Supplementary Table 5. Life cycle inventory[a].

Impact Category	Water consumption (m^3)	Energy consumption (MJ)	References
Conductive carbon (Metcoke)	0.422	28017	GREET
Water	1	0	GREET
H_2O_2 (30%)	1.9046	15865	GREET
1-pentanol	34.4	46627	GREET[b]
ethanol	34.4	46627	GREET
Mixing (solid-solid)	0	9.432	Estimated
HET process	0	1512	This work
Sieving	0	4	Estimated
Furnace heating	0	2800	Estimated
Agitation	0	2.88	Estimated
Filtration	0	2.2	Estimated

Note: [a]The environmental impacts or energy demands are normalized to production or processing of 1 tonne of materials. [b]Ethanol was used as a proxy for 1-pentanol because 1-pentanol was not included in the GREET database.

Supplementary Table 6. Cumulative water use (CWU) for various scenarios.

Scenarios	HET (tonne)	Thermal desorption (tonne)	Soil washing (tonne)	Chemical degradation (tonne)
Conductive carbon (Metcoke)	0.01688	0	0	0
Water	0	0	1.2	85.5
H_2O_2 (30%)	0	0	0	0.6132
1-pentanol	0	0	0.3357	0
ethanol	0	0	5.537	0
SUM of Materials	0.017	0	7.07	86.11
Mixing (solid-solid)	0	0	0	0
HET process	0	0	0	0
Sieving	0	0	0	0
Furnace heating	0	0	0	0
Agitation	0	0	0	0
Filtration	0	0	0	0
SUM of Process	0	0	7.07	0
SUM	0.017	0	7.07	86.11

Note: [a]The materials mass flow is normalized to remediation of 1 tonne of PAH-contaminated soil.

Supplementary Table 7. Cumulative energy demand for various scenarios.

Scenarios	HET (tonne)	Thermal desorption (tonne)	Soil washing (tonne)	Chemical degradation (tonne)
Conductive carbon (Metcoke)	1120.68	0	0	0
Water	0	0	0	0
H_2O_2 (30%)	0	0	0	5108.53
1-pentanol	0	0	455.08	0
ethanol	0	0	7505.08	0
SUM of Materials	1120.68	0	7960.16	5108.53
Mixing (solid-solid)	14.148	0	0	0
HET process	2268	0	0	0
Sieving	5.34	0	0	0
Furnace heating	0	2800	0	0
Agitation	0	0	36.68	258.39
Filtration	0	0	28.02	197.384
SUM of Process	2287.49	2800	64.70	455.77
SUM	3408.17	2800	8013.60	5564.30

Note: [a]The materials mass flow is normalized to remediation of 1 tonne of PAH-contaminated soil.

Supplementary Table 8. Materials and energy expense inventory.

Impact Category	Materials expense ($)	Energy expense ($)
Conductive carbon (Metcoke)	150	0
Water	0.5	0
H_2O_2 (30%)	350	0
1-pentanol	1000	0
ethanol	800	0
Mixing (solid-solid)	0	0.154
HET process	0	24.656
Sieving	0	0.065
Furnace heating	0	45.66
Agitation	0	0.047
Filtration	0	0.036

[a]The materials and energy expense are normalized to production or processing of 1 tonne of materials.

Supplementary Table 9. Expense evaluation for various scenarios.

Scenarios	HET ($)	Thermal desorption ($)	Soil washing ($)	Chemical degradation ($)
Conductive carbon (Metcoke)	6	0	0	0
Water	0	0	0.6	42.75
H_2O_2 (30%)	0	0	0	112.7
1-pentanol	0	0	9.76	0
ethanol	0	0	128.768	0
**SUM of Materials**	6	0	139.128	155.45
Mixing (solid-solid)	0.231	0	0	0
HET process	36.984	0	0	0
Sieving	0.0867	0	0	0
Furnace heating	0	45.66	0	0
Agitation	0	0	0.599	4.217
Filtration	0	0	0.458	3.30
**SUM of Energy**	37.30	45.66	1.057	7.517
**Operating Expense**	43.30	45.66	140.19	162.97
**Capital Expense**	8.35 – 16.7	---	---	9 – 18
**Total Expense**	51.7 – 60	46 – 99	50 – 165	172 – 181

Note: [a]The materials mass flow is normalized to remediation of 1 tonne of PAH-contaminated soil.

Supplementary References

1 Wang, L. *et al.* Remediation of mercury contaminated soil, water, and air: A review of emerging materials and innovative technologies. *Environ. Int.* **134**, 105281 (2020).

2 Wieland, K. Thermal decomposition of $HgCl_2$ vapour. *Nature* **156**, 504-505 (1945).

3 L'Vov, B. V. Kinetics and mechanism of thermal decomposition of mercuric oxide. *Thermochim. Acta* **333**, 21-26 (1999).

4 Tariq, S. A. & Hill, J. O. Thermal analysis of mercury(I) sulfate and mercury(II) sulfate. *J. Therm. Anal.* **21**, 277-281 (1981).

5 Xu, X.-Y., Hu, N., Wang, Q., Fan, L.-W. & Song, X. A numerical study of optimizing the well spacing and heating power for in situ thermal remediation of organic-contaminated soil. *Case Stud. Therm. Eng.* **33**, 101941 (2022).

6 Lemming, G. *et al.* Optimizing the environmental performance of in situ thermal remediation technologies using life cycle assessment. *Ground Water Monit. Remediat.* **33**, 38-51 (2013).

7 Streche, C., Cocârţă, D. M., Istrate, I.-A. & Badea, A. A. Decontamination of petroleum-contaminated soils using the electrochemical technique: remediation degree and energy consumption. *Sci. Rep.* **8**, 3272 (2018).

8 International Organization for Standardization (ISO), The New International Standards for Life Cycle Assessment: ISO 14040 and ISO 14044 (ISO, Geneva, Switzerland, 2006).

9 Kumar, M. *et al.* Remediation of soils and sediments polluted with polycyclic aromatic hydrocarbons: To immobilize, mobilize, or degrade? *J. Hazard. Mater.* **420**, 126534 (2021).

10 Choi, B., Lee, S. & Jho, E. H. Removal of TPH, UCM, PAHs, and Alk-PAHs in oil-contaminated soil by thermal desorption. *Appl. Biol. Chem.* **63**, 83 (2020).

11 Khodadoust, A. P., Bagchi, R., Suidan, M. T., Brenner, R. C. & Sellers, N. G. Removal of PAHs from highly contaminated soils found at prior manufactured gas operations. *J. Hazard. Mater.* **80**, 159-174 (2000).

12 Biache, C., Lorgeoux, C., Andriatsihoarana, S., Colombano, S. & Faure, P. Effect of pre-heating on the chemical oxidation efficiency: Implications for the PAH availability measurement in contaminated soils. *J. Hazard. Mater.* **286**, 55-63 (2015).

13 Tatàno, F., Felici, F. & Mangani, F. Lab-scale treatability tests for the thermal desorption of hydrocarbon-contaminated soils. *Soil Sediment Contam.* **22**, 433-456 (2013).

14 https://greet.es.anl.gov/

15 https://www.lfatabletpresses.com/vh-powder-mixer, accessed July 10th, 2023

16 https://www.carbolite-gero.com/products/chamber-furnaces/industrial-furnaces/sbcf/, accessed 10th July.

17 https://www.agitadoresfluidmix.com/en/industrial-agitator-vpp/, accessed 10th July.

18 https://www.alibaba.com/product-detail/Metallurgical-10-80mm-Metallurgical-Coke-Price_1600716599553.html?spm=a2700.galleryofferlist.normal_offer.d_title.4b5e34cevz7bt9&s=p, accessed 10th July.

19 https://www.fbgtx.org/673/Industrial-Water-Rates, accessed 10th July.

20 https://ruisunny-chem.en.made-in-china.com/product/ydFaTZrxZNpn/China-Industrial-Grade-Hydrogen-Peroxide-Liquid-35-50-IBC-Tank.html, accessed 10th July.

21 https://guanlangbio1.en.made-in-china.com/product/MmAYJRpPohkE/China-High-Quality-1-Pentanol-CAS-71-41-0-in-Stock.html, accessed 10th July.

22 https://9dc0dfc9de832ae4.en.made-in-china.com/product/jOaTnwmoAbRd/China-Hot-Sale-99-Purity-Ethanol-CAS-64-17-5.html, accessed 10th July.

23 https://www.eia.gov/electricity/monthly/epm_table_grapher.php?t=epmt_5_6_a, accessed 10th July.

24 Vidonish, J. E., Zygourakis, K., Masiello, C. A., Sabadell, G. & Alvarez, P. J. J. Thermal treatment of hydrocarbon-impacted soils: A review of technology innovation for sustainable remediation. *Engineering* **2**, 426-437 (2016).

25 Soil washing remedial alternative screening technical memorandum for USS lead OU1 zone 1 site east Chicago, Indiana, US EPA, 2018.

26 https://www.alibaba.com/product-detail/Agitator-Mixer-Stainless-Steel-Tank-Mixer_60616253974.html?s=p, accessed on 11st July, 2023.

27 Luong, D. X. *et al.* Gram-scale bottom-up flash graphene synthesis. *Nature* **577**, 647-651 (2020).

28 Stanford, M. G. *et al.* Flash Graphene Morphologies. *ACS Nano* **14**, 13691-13699 (2020).

29 Chekol, T., Vough, L. R. & Chaney, R. L. Phytoremediation of polychlorinated biphenyl-contaminated soils: the rhizosphere effect. *Environ. Int.* **30**, 799-804 (2004).

30 M. Makoudi, A. Jordens, and J. Haemers, In Situ Thermal Desorption using Smart Burners technology in urban area for the treatment of -VOCl- contaminated soil, 2021, HAEMERS Technologies
